# Measurement of the top quark mass using single top quark events in proton-proton collisions at $\sqrt{s}= 8$ TeV

**DOI:** 10.1140/epjc/s10052-017-4912-8

**Published:** 2017-05-29

**Authors:** A. M. Sirunyan, A. Tumasyan, W. Adam, E. Asilar, T. Bergauer, J. Brandstetter, E. Brondolin, M. Dragicevic, J. Erö, M. Flechl, M. Friedl, R. Frühwirth, V. M. Ghete, C. Hartl, N. Hörmann, J. Hrubec, M. Jeitler, A. König, I. Krätschmer, D. Liko, T. Matsushita, I. Mikulec, D. Rabady, N. Rad, B. Rahbaran, H. Rohringer, J. Schieck, J. Strauss, W. Waltenberger, C.-E. Wulz, O. Dvornikov, V. Makarenko, V. Mossolov, J. Suarez Gonzalez, V. Zykunov, N. Shumeiko, S. Alderweireldt, E. A. De Wolf, X. Janssen, J. Lauwers, M. Van De Klundert, H. Van Haevermaet, P. Van Mechelen, N. Van Remortel, A. Van Spilbeeck, S. Abu Zeid, F. Blekman, J. D’Hondt, N. Daci, I. De Bruyn, K. Deroover, S. Lowette, S. Moortgat, L. Moreels, A. Olbrechts, Q. Python, K. Skovpen, S. Tavernier, W. Van Doninck, P. Van Mulders, I. Van Parijs, H. Brun, B. Clerbaux, G. De Lentdecker, H. Delannoy, G. Fasanella, L. Favart, R. Goldouzian, A. Grebenyuk, G. Karapostoli, T. Lenzi, A. Léonard, J. Luetic, T. Maerschalk, A. Marinov, A. Randle-conde, T. Seva, C. Vander Velde, P. Vanlaer, D. Vannerom, R. Yonamine, F. Zenoni, F. Zhang, A. Cimmino, T. Cornelis, D. Dobur, A. Fagot, M. Gul, I. Khvastunov, D. Poyraz, S. Salva, R. Schöfbeck, M. Tytgat, W. Van Driessche, E. Yazgan, N. Zaganidis, H. Bakhshiansohi, C. Beluffi, O. Bondu, S. Brochet, G. Bruno, A. Caudron, S. De Visscher, C. Delaere, M. Delcourt, B. Francois, A. Giammanco, A. Jafari, M. Komm, G. Krintiras, V. Lemaitre, A. Magitteri, A. Mertens, M. Musich, K. Piotrzkowski, L. Quertenmont, M. Selvaggi, M. Vidal Marono, S. Wertz, N. Beliy, W. L. Aldá Júnior, F. L. Alves, G. A. Alves, L. Brito, C. Hensel, A. Moraes, M. E. Pol, P. Rebello Teles, E. Belchior Batista Das Chagas, W. Carvalho, J. Chinellato, A. Custódio, E. M. Da Costa, G. G. Da Silveira, D. De Jesus Damiao, C. De Oliveira Martins, S. Fonseca De Souza, L. M. Huertas Guativa, H. Malbouisson, D. Matos Figueiredo, C. Mora Herrera, L. Mundim, H. Nogima, W. L. Prado Da Silva, A. Santoro, A. Sznajder, E. J. Tonelli Manganote, F. Torres Da Silva De Araujo, A. Vilela Pereira, S. Ahuja, C. A. Bernardes, S. Dogra, T. R. Fernandez Perez Tomei, E. M. Gregores, P. G. Mercadante, C. S. Moon, S. F. Novaes, Sandra S. Padula, D. Romero Abad, J. C. Ruiz Vargas, A. Aleksandrov, R. Hadjiiska, P. Iaydjiev, M. Rodozov, S. Stoykova, G. Sultanov, M. Vutova, A. Dimitrov, I. Glushkov, L. Litov, B. Pavlov, P. Petkov, W. Fang, M. Ahmad, J. G. Bian, G. M. Chen, H. S. Chen, M. Chen, Y. Chen, T. Cheng, C. H. Jiang, D. Leggat, Z. Liu, F. Romeo, M. Ruan, S. M. Shaheen, A. Spiezia, J. Tao, C. Wang, Z. Wang, H. Zhang, J. Zhao, Y. Ban, G. Chen, Q. Li, S. Liu, Y. Mao, S. J. Qian, D. Wang, Z. Xu, C. Avila, A. Cabrera, L. F. Chaparro Sierra, C. Florez, J. P. Gomez, C. F. González Hernández, J. D. Ruiz Alvarez, J. C. Sanabria, N. Godinovic, D. Lelas, I. Puljak, P. M. Ribeiro Cipriano, T. Sculac, Z. Antunovic, M. Kovac, V. Brigljevic, D. Ferencek, K. Kadija, B. Mesic, T. Susa, A. Attikis, G. Mavromanolakis, J. Mousa, C. Nicolaou, F. Ptochos, P. A. Razis, H. Rykaczewski, D. Tsiakkouri, M. Finger, M. Finger, E. Carrera Jarrin, E. El-khateeb, S. Elgammal, A. Mohamed, M. Kadastik, L. Perrini, M. Raidal, A. Tiko, C. Veelken, P. Eerola, J. Pekkanen, M. Voutilainen, J. Härkönen, T. Järvinen, V. Karimäki, R. Kinnunen, T. Lampén, K. Lassila-Perini, S. Lehti, T. Lindén, P. Luukka, J. Tuominiemi, E. Tuovinen, L. Wendland, J. Talvitie, T. Tuuva, M. Besancon, F. Couderc, M. Dejardin, D. Denegri, B. Fabbro, J. L. Faure, C. Favaro, F. Ferri, S. Ganjour, S. Ghosh, A. Givernaud, P. Gras, G. Hamel de Monchenault, P. Jarry, I. Kucher, E. Locci, M. Machet, J. Malcles, J. Rander, A. Rosowsky, M. Titov, A. Abdulsalam, I. Antropov, S. Baffioni, F. Beaudette, P. Busson, L. Cadamuro, E. Chapon, C. Charlot, O. Davignon, R. Granier de Cassagnac, M. Jo, S. Lisniak, P. Miné, M. Nguyen, C. Ochando, G. Ortona, P. Paganini, P. Pigard, S. Regnard, R. Salerno, Y. Sirois, A. G. Stahl Leiton, T. Strebler, Y. Yilmaz, A. Zabi, A. Zghiche, J.-L. Agram, J. Andrea, A. Aubin, D. Bloch, J.-M. Brom, M. Buttignol, E. C. Chabert, N. Chanon, C. Collard, E. Conte, X. Coubez, J.-C. Fontaine, D. Gelé, U. Goerlach, A.-C. Le Bihan, P. Van Hove, S. Gadrat, S. Beauceron, C. Bernet, G. Boudoul, C. A. Carrillo Montoya, R. Chierici, D. Contardo, B. Courbon, P. Depasse, H. El Mamouni, J. Fay, S. Gascon, M. Gouzevitch, G. Grenier, B. Ille, F. Lagarde, I. B. Laktineh, M. Lethuillier, L. Mirabito, A. L. Pequegnot, S. Perries, A. Popov, D. Sabes, V. Sordini, M. Vander Donckt, P. Verdier, S. Viret, A. Khvedelidze, Z. Tsamalaidze, C. Autermann, S. Beranek, L. Feld, M. K. Kiesel, K. Klein, M. Lipinski, M. Preuten, C. Schomakers, J. Schulz, T. Verlage, A. Albert, M. Brodski, E. Dietz-Laursonn, D. Duchardt, M. Endres, M. Erdmann, S. Erdweg, T. Esch, R. Fischer, A. Güth, M. Hamer, T. Hebbeker, C. Heidemann, K. Hoepfner, S. Knutzen, M. Merschmeyer, A. Meyer, P. Millet, S. Mukherjee, M. Olschewski, K. Padeken, T. Pook, M. Radziej, H. Reithler, M. Rieger, F. Scheuch, L. Sonnenschein, D. Teyssier, S. Thüer, V. Cherepanov, G. Flügge, B. Kargoll, T. Kress, A. Künsken, J. Lingemann, T. Müller, A. Nehrkorn, A. Nowack, C. Pistone, O. Pooth, A. Stahl, M. Aldaya Martin, T. Arndt, C. Asawatangtrakuldee, K. Beernaert, O. Behnke, U. Behrens, A. A. Bin Anuar, K. Borras, A. Campbell, P. Connor, C. Contreras-Campana, F. Costanza, C. Diez Pardos, G. Dolinska, G. Eckerlin, D. Eckstein, T. Eichhorn, E. Eren, E. Gallo, J. Garay Garcia, A. Geiser, A. Gizhko, J. M. Grados Luyando, A. Grohsjean, P. Gunnellini, A. Harb, J. Hauk, M. Hempel, H. Jung, A. Kalogeropoulos, O. Karacheban, M. Kasemann, J. Keaveney, C. Kleinwort, I. Korol, D. Krücker, W. Lange, A. Lelek, T. Lenz, J. Leonard, K. Lipka, A. Lobanov, W. Lohmann, R. Mankel, I.-A. Melzer-Pellmann, A. B. Meyer, G. Mittag, J. Mnich, A. Mussgiller, D. Pitzl, R. Placakyte, A. Raspereza, B. Roland, M. Ö. Sahin, P. Saxena, T. Schoerner-Sadenius, S. Spannagel, N. Stefaniuk, G. P. Van Onsem, R. Walsh, C. Wissing, V. Blobel, M. Centis Vignali, A. R. Draeger, T. Dreyer, E. Garutti, D. Gonzalez, J. Haller, M. Hoffmann, A. Junkes, R. Klanner, R. Kogler, N. Kovalchuk, T. Lapsien, I. Marchesini, D. Marconi, M. Meyer, M. Niedziela, D. Nowatschin, F. Pantaleo, T. Peiffer, A. Perieanu, C. Scharf, P. Schleper, A. Schmidt, S. Schumann, J. Schwandt, H. Stadie, G. Steinbrück, F. M. Stober, M. Stöver, H. Tholen, D. Troendle, E. Usai, L. Vanelderen, A. Vanhoefer, B. Vormwald, M. Akbiyik, C. Barth, S. Baur, C. Baus, J. Berger, E. Butz, R. Caspart, T. Chwalek, F. Colombo, W. De Boer, A. Dierlamm, S. Fink, B. Freund, R. Friese, M. Giffels, A. Gilbert, P. Goldenzweig, D. Haitz, F. Hartmann, S. M. Heindl, U. Husemann, I. Katkov, S. Kudella, H. Mildner, M. U. Mozer, Th. Müller, M. Plagge, G. Quast, K. Rabbertz, S. Röcker, F. Roscher, M. Schröder, I. Shvetsov, G. Sieber, H. J. Simonis, R. Ulrich, S. Wayand, M. Weber, T. Weiler, S. Williamson, C. Wöhrmann, R. Wolf, G. Anagnostou, G. Daskalakis, T. Geralis, V. A. Giakoumopoulou, A. Kyriakis, D. Loukas, I. Topsis-Giotis, S. Kesisoglou, A. Panagiotou, N. Saoulidou, E. Tziaferi, I. Evangelou, G. Flouris, C. Foudas, P. Kokkas, N. Loukas, N. Manthos, I. Papadopoulos, E. Paradas, N. Filipovic, G. Pasztor, G. Bencze, C. Hajdu, D. Horvath, F. Sikler, V. Veszpremi, G. Vesztergombi, A. J. Zsigmond, N. Beni, S. Czellar, J. Karancsi, A. Makovec, J. Molnar, Z. Szillasi, M. Bartók, P. Raics, Z. L. Trocsanyi, B. Ujvari, J. R. Komaragiri, S. Bahinipati, S. Bhowmik, S. Choudhury, P. Mal, K. Mandal, A. Nayak, D. K. Sahoo, N. Sahoo, S. K. Swain, S. Bansal, S. B. Beri, V. Bhatnagar, R. Chawla, U. Bhawandeep, A. K. Kalsi, A. Kaur, M. Kaur, R. Kumar, P. Kumari, A. Mehta, M. Mittal, J. B. Singh, G. Walia, Ashok Kumar, A. Bhardwaj, B. C. Choudhary, R. B. Garg, S. Keshri, S. Malhotra, M. Naimuddin, K. Ranjan, R. Sharma, V. Sharma, R. Bhattacharya, S. Bhattacharya, K. Chatterjee, S. Dey, S. Dutt, S. Dutta, S. Ghosh, N. Majumdar, A. Modak, K. Mondal, S. Mukhopadhyay, S. Nandan, A. Purohit, A. Roy, D. Roy, S. Roy Chowdhury, S. Sarkar, M. Sharan, S. Thakur, P. K. Behera, R. Chudasama, D. Dutta, V. Jha, V. Kumar, A. K. Mohanty, P. K. Netrakanti, L. M. Pant, P. Shukla, A. Topkar, T. Aziz, S. Dugad, G. Kole, B. Mahakud, S. Mitra, G. B. Mohanty, B. Parida, N. Sur, B. Sutar, S. Banerjee, R. K. Dewanjee, S. Ganguly, M. Guchait, Sa. Jain, S. Kumar, M. Maity, G. Majumder, K. Mazumdar, T. Sarkar, N. Wickramage, S. Chauhan, S. Dube, V. Hegde, A. Kapoor, K. Kothekar, S. Pandey, A. Rane, S. Sharma, S. Chenarani, E. Eskandari Tadavani, S. M. Etesami, M. Khakzad, M. Mohammadi Najafabadi, M. Naseri, S. Paktinat Mehdiabadi, F. Rezaei Hosseinabadi, B. Safarzadeh, M. Zeinali, M. Felcini, M. Grunewald, M. Abbrescia, C. Calabria, C. Caputo, A. Colaleo, D. Creanza, L. Cristella, N. De Filippis, M. De Palma, L. Fiore, G. Iaselli, G. Maggi, M. Maggi, G. Miniello, S. My, S. Nuzzo, A. Pompili, G. Pugliese, R. Radogna, A. Ranieri, G. Selvaggi, A. Sharma, L. Silvestris, R. Venditti, P. Verwilligen, G. Abbiendi, C. Battilana, D. Bonacorsi, S. Braibant-Giacomelli, L. Brigliadori, R. Campanini, P. Capiluppi, A. Castro, F. R. Cavallo, S. S. Chhibra, G. Codispoti, M. Cuffiani, G. M. Dallavalle, F. Fabbri, A. Fanfani, D. Fasanella, P. Giacomelli, C. Grandi, L. Guiducci, S. Marcellini, G. Masetti, A. Montanari, F. L. Navarria, A. Perrotta, A. M. Rossi, T. Rovelli, G. P. Siroli, N. Tosi, S. Albergo, S. Costa, A. Di Mattia, F. Giordano, R. Potenza, A. Tricomi, C. Tuve, G. Barbagli, V. Ciulli, C. Civinini, R. D’Alessandro, E. Focardi, P. Lenzi, M. Meschini, S. Paoletti, L. Russo, G. Sguazzoni, D. Strom, L. Viliani, L. Benussi, S. Bianco, F. Fabbri, D. Piccolo, F. Primavera, V. Calvelli, F. Ferro, M. R. Monge, E. Robutti, S. Tosi, L. Brianza, F. Brivio, V. Ciriolo, M. E. Dinardo, S. Fiorendi, S. Gennai, A. Ghezzi, P. Govoni, M. Malberti, S. Malvezzi, R. A. Manzoni, D. Menasce, L. Moroni, M. Paganoni, D. Pedrini, S. Pigazzini, S. Ragazzi, T. Tabarelli de Fatis, S. Buontempo, N. Cavallo, G. De Nardo, S. Di Guida, M. Esposito, F. Fabozzi, F. Fienga, A. O. M. Iorio, G. Lanza, L. Lista, S. Meola, P. Paolucci, C. Sciacca, F. Thyssen, P. Azzi, N. Bacchetta, L. Benato, D. Bisello, A. Boletti, R. Carlin, A. Carvalho Antunes De Oliveira, P. Checchia, M. Dall’Osso, P. De Castro Manzano, T. Dorigo, U. Dosselli, F. Gasparini, U. Gasparini, A. Gozzelino, S. Lacaprara, M. Margoni, A. T. Meneguzzo, J. Pazzini, N. Pozzobon, P. Ronchese, F. Simonetto, E. Torassa, M. Zanetti, P. Zotto, G. Zumerle, A. Braghieri, F. Fallavollita, A. Magnani, P. Montagna, S. P. Ratti, V. Re, C. Riccardi, P. Salvini, I. Vai, P. Vitulo, L. Alunni Solestizi, G. M. Bilei, D. Ciangottini, L. Fanò, P. Lariccia, R. Leonardi, G. Mantovani, M. Menichelli, A. Saha, A. Santocchia, K. Androsov, P. Azzurri, G. Bagliesi, J. Bernardini, T. Boccali, R. Castaldi, M. A. Ciocci, R. Dell’Orso, S. Donato, G. Fedi, A. Giassi, M. T. Grippo, F. Ligabue, T. Lomtadze, L. Martini, A. Messineo, F. Palla, A. Rizzi, A. Savoy-Navarro, P. Spagnolo, R. Tenchini, G. Tonelli, A. Venturi, P. G. Verdini, L. Barone, F. Cavallari, M. Cipriani, D. Del Re, M. Diemoz, S. Gelli, E. Longo, F. Margaroli, B. Marzocchi, P. Meridiani, G. Organtini, R. Paramatti, F. Preiato, S. Rahatlou, C. Rovelli, F. Santanastasio, N. Amapane, R. Arcidiacono, S. Argiro, M. Arneodo, N. Bartosik, R. Bellan, C. Biino, N. Cartiglia, F. Cenna, M. Costa, R. Covarelli, A. Degano, N. Demaria, L. Finco, B. Kiani, C. Mariotti, S. Maselli, E. Migliore, V. Monaco, E. Monteil, M. Monteno, M. M. Obertino, L. Pacher, N. Pastrone, M. Pelliccioni, G. L. Pinna Angioni, F. Ravera, A. Romero, M. Ruspa, R. Sacchi, K. Shchelina, V. Sola, A. Solano, A. Staiano, P. Traczyk, S. Belforte, M. Casarsa, F. Cossutti, G. Della Ricca, A. Zanetti, D. H. Kim, G. N. Kim, M. S. Kim, S. Lee, S. W. Lee, Y. D. Oh, S. Sekmen, D. C. Son, Y. C. Yang, A. Lee, H. Kim, J. A. Brochero Cifuentes, T. J. Kim, S. Cho, S. Choi, Y. Go, D. Gyun, S. Ha, B. Hong, Y. Jo, Y. Kim, K. Lee, K. S. Lee, S. Lee, J. Lim, S. K. Park, Y. Roh, J. Almond, J. Kim, H. Lee, S. B. Oh, B. C. Radburn-Smith, S. h. Seo, U. K. Yang, H. D. Yoo, G. B. Yu, M. Choi, H. Kim, J. H. Kim, J. S. H. Lee, I. C. Park, G. Ryu, M. S. Ryu, Y. Choi, J. Goh, C. Hwang, J. Lee, I. Yu, V. Dudenas, A. Juodagalvis, J. Vaitkus, I. Ahmed, Z. A. Ibrahim, M. A. B. Md Ali, F. Mohamad Idris, W. A. T. Wan Abdullah, M. N. Yusli, Z. Zolkapli, H. Castilla-Valdez, E. De La Cruz-Burelo, I. Heredia-De La Cruz, A. Hernandez-Almada, R. Lopez-Fernandez, R. Magaña Villalba, J. Mejia Guisao, A. Sanchez-Hernandez, S. Carrillo Moreno, C. Oropeza Barrera, F. Vazquez Valencia, S. Carpinteyro, I. Pedraza, H. A. Salazar Ibarguen, C. Uribe Estrada, A. Morelos Pineda, D. Krofcheck, P. H. Butler, A. Ahmad, M. Ahmad, Q. Hassan, H. R. Hoorani, W. A. Khan, A. Saddique, M. A. Shah, M. Shoaib, M. Waqas, H. Bialkowska, M. Bluj, B. Boimska, T. Frueboes, M. Górski, M. Kazana, K. Nawrocki, K. Romanowska-Rybinska, M. Szleper, P. Zalewski, K. Bunkowski, A. Byszuk, K. Doroba, A. Kalinowski, M. Konecki, J. Krolikowski, M. Misiura, M. Olszewski, M. Walczak, P. Bargassa, C. Beirão Da Cruz E Silva, B. Calpas, A. Di Francesco, P. Faccioli, P. G. Ferreira Parracho, M. Gallinaro, J. Hollar, N. Leonardo, L. Lloret Iglesias, M. V. Nemallapudi, J. Rodrigues Antunes, J. Seixas, O. Toldaiev, D. Vadruccio, J. Varela, S. Afanasiev, P. Bunin, M. Gavrilenko, I. Golutvin, I. Gorbunov, A. Kamenev, V. Karjavin, A. Lanev, A. Malakhov, V. Matveev, V. Palichik, V. Perelygin, S. Shmatov, S. Shulha, N. Skatchkov, V. Smirnov, N. Voytishin, A. Zarubin, L. Chtchipounov, V. Golovtsov, Y. Ivanov, V. Kim, E. Kuznetsova, V. Murzin, V. Oreshkin, V. Sulimov, A. Vorobyev, Yu. Andreev, A. Dermenev, S. Gninenko, N. Golubev, A. Karneyeu, M. Kirsanov, N. Krasnikov, A. Pashenkov, D. Tlisov, A. Toropin, V. Epshteyn, V. Gavrilov, N. Lychkovskaya, V. Popov, I. Pozdnyakov, G. Safronov, A. Spiridonov, M. Toms, E. Vlasov, A. Zhokin, T. Aushev, A. Bylinkin, M. Danilov, E. Popova, V. Rusinov, V. Andreev, M. Azarkin, I. Dremin, M. Kirakosyan, A. Leonidov, A. Terkulov, A. Baskakov, A. Belyaev, E. Boos, V. Bunichev, M. Dubinin, L. Dudko, V. Klyukhin, O. Kodolova, N. Korneeva, I. Lokhtin, I. Miagkov, S. Obraztsov, M. Perfilov, V. Savrin, P. Volkov, V. Blinov, Y. Skovpen, D. Shtol, I. Azhgirey, I. Bayshev, S. Bitioukov, D. Elumakhov, V. Kachanov, A. Kalinin, D. Konstantinov, V. Krychkine, V. Petrov, R. Ryutin, A. Sobol, S. Troshin, N. Tyurin, A. Uzunian, A. Volkov, P. Adzic, P. Cirkovic, D. Devetak, M. Dordevic, J. Milosevic, V. Rekovic, J. Alcaraz Maestre, M. Barrio Luna, E. Calvo, M. Cerrada, M. Chamizo Llatas, N. Colino, B. De La Cruz, A. Delgado Peris, A. Escalante Del Valle, C. Fernandez Bedoya, J. P. Fernández Ramos, J. Flix, M. C. Fouz, P. Garcia-Abia, O. Gonzalez Lopez, S. Goy Lopez, J. M. Hernandez, M. I. Josa, E. Navarro De Martino, A. Pérez-Calero Yzquierdo, J. Puerta Pelayo, A. Quintario Olmeda, I. Redondo, L. Romero, M. S. Soares, J. F. de Trocóniz, M. Missiroli, D. Moran, J. Cuevas, J. Fernandez Menendez, I. Gonzalez Caballero, J. R. González Fernández, E. Palencia Cortezon, S. Sanchez Cruz, I. Suárez Andrés, P. Vischia, J. M. Vizan Garcia, I. J. Cabrillo, A. Calderon, E. Curras, M. Fernandez, J. Garcia-Ferrero, G. Gomez, A. Lopez Virto, J. Marco, C. Martinez Rivero, F. Matorras, J. Piedra Gomez, T. Rodrigo, A. Ruiz-Jimeno, L. Scodellaro, N. Trevisani, I. Vila, R. Vilar Cortabitarte, D. Abbaneo, E. Auffray, G. Auzinger, P. Baillon, A. H. Ball, D. Barney, P. Bloch, A. Bocci, C. Botta, T. Camporesi, R. Castello, M. Cepeda, G. Cerminara, Y. Chen, D. d’Enterria, A. Dabrowski, V. Daponte, A. David, M. De Gruttola, A. De Roeck, E. Di Marco, M. Dobson, B. Dorney, T. du Pree, D. Duggan, M. Dünser, N. Dupont, A. Elliott-Peisert, P. Everaerts, S. Fartoukh, G. Franzoni, J. Fulcher, W. Funk, D. Gigi, K. Gill, M. Girone, F. Glege, D. Gulhan, S. Gundacker, M. Guthoff, P. Harris, J. Hegeman, V. Innocente, P. Janot, J. Kieseler, H. Kirschenmann, V. Knünz, A. Kornmayer, M. J. Kortelainen, K. Kousouris, M. Krammer, C. Lange, P. Lecoq, C. Lourenço, M. T. Lucchini, L. Malgeri, M. Mannelli, A. Martelli, F. Meijers, J. A. Merlin, S. Mersi, E. Meschi, P. Milenovic, F. Moortgat, S. Morovic, M. Mulders, H. Neugebauer, S. Orfanelli, L. Orsini, L. Pape, E. Perez, M. Peruzzi, A. Petrilli, G. Petrucciani, A. Pfeiffer, M. Pierini, A. Racz, T. Reis, G. Rolandi, M. Rovere, H. Sakulin, J. B. Sauvan, C. Schäfer, C. Schwick, M. Seidel, A. Sharma, P. Silva, P. Sphicas, J. Steggemann, M. Stoye, Y. Takahashi, M. Tosi, D. Treille, A. Triossi, A. Tsirou, V. Veckalns, G. I. Veres, M. Verweij, N. Wardle, H. K. Wöhri, A. Zagozdzinska, W. D. Zeuner, W. Bertl, K. Deiters, W. Erdmann, R. Horisberger, Q. Ingram, H. C. Kaestli, D. Kotlinski, U. Langenegger, T. Rohe, S. A. Wiederkehr, F. Bachmair, L. Bäni, L. Bianchini, B. Casal, G. Dissertori, M. Dittmar, M. Donegà, C. Grab, C. Heidegger, D. Hits, J. Hoss, G. Kasieczka, W. Lustermann, B. Mangano, M. Marionneau, P. Martinez Ruiz del Arbol, M. Masciovecchio, M. T. Meinhard, D. Meister, F. Micheli, P. Musella, F. Nessi-Tedaldi, F. Pandolfi, J. Pata, F. Pauss, G. Perrin, L. Perrozzi, M. Quittnat, M. Rossini, M. Schönenberger, A. Starodumov, V. R. Tavolaro, K. Theofilatos, R. Wallny, T. K. Aarrestad, C. Amsler, L. Caminada, M. F. Canelli, A. De Cosa, C. Galloni, A. Hinzmann, T. Hreus, B. Kilminster, J. Ngadiuba, D. Pinna, G. Rauco, P. Robmann, D. Salerno, C. Seitz, Y. Yang, A. Zucchetta, V. Candelise, T. H. Doan, Sh. Jain, R. Khurana, M. Konyushikhin, C. M. Kuo, W. Lin, A. Pozdnyakov, S. S. Yu, Arun Kumar, P. Chang, Y. H. Chang, Y. Chao, K. F. Chen, P. H. Chen, F. Fiori, W.-S. Hou, Y. Hsiung, Y. F. Liu, R.-S. Lu, M. Miñano Moya, E. Paganis, A. Psallidas, J. F. Tsai, B. Asavapibhop, G. Singh, N. Srimanobhas, N. Suwonjandee, A. Adiguzel, S. Damarseckin, Z. S. Demiroglu, C. Dozen, E. Eskut, S. Girgis, G. Gokbulut, Y. Guler, I. Hos, E. E. Kangal, O. Kara, A. Kayis Topaksu, U. Kiminsu, M. Oglakci, G. Onengut, K. Ozdemir, S. Ozturk, A. Polatoz, B. Tali, S. Turkcapar, I. S. Zorbakir, C. Zorbilmez, B. Bilin, S. Bilmis, B. Isildak, G. Karapinar, M. Yalvac, M. Zeyrek, E. Gülmez, M. Kaya, O. Kaya, E. A. Yetkin, T. Yetkin, A. Cakir, K. Cankocak, S. Sen, B. Grynyov, L. Levchuk, P. Sorokin, R. Aggleton, F. Ball, L. Beck, J. J. Brooke, D. Burns, E. Clement, D. Cussans, H. Flacher, J. Goldstein, M. Grimes, G. P. Heath, H. F. Heath, J. Jacob, L. Kreczko, C. Lucas, D. M. Newbold, S. Paramesvaran, A. Poll, T. Sakuma, S. Seif El Nasr-storey, D. Smith, V. J. Smith, K. W. Bell, A. Belyaev, C. Brew, R. M. Brown, L. Calligaris, D. Cieri, D. J. A. Cockerill, J. A. Coughlan, K. Harder, S. Harper, E. Olaiya, D. Petyt, C. H. Shepherd-Themistocleous, A. Thea, I. R. Tomalin, T. Williams, M. Baber, R. Bainbridge, O. Buchmuller, A. Bundock, D. Burton, S. Casasso, M. Citron, D. Colling, L. Corpe, P. Dauncey, G. Davies, A. De Wit, M. Della Negra, R. Di Maria, P. Dunne, A. Elwood, D. Futyan, Y. Haddad, G. Hall, G. Iles, T. James, R. Lane, C. Laner, R. Lucas, L. Lyons, A.-M. Magnan, S. Malik, L. Mastrolorenzo, J. Nash, A. Nikitenko, J. Pela, B. Penning, M. Pesaresi, D. M. Raymond, A. Richards, A. Rose, E. Scott, C. Seez, S. Summers, A. Tapper, K. Uchida, M. Vazquez Acosta, T. Virdee, J. Wright, S. C. Zenz, J. E. Cole, P. R. Hobson, A. Khan, P. Kyberd, I. D. Reid, P. Symonds, L. Teodorescu, M. Turner, A. Borzou, K. Call, J. Dittmann, K. Hatakeyama, H. Liu, N. Pastika, R. Bartek, A. Dominguez, A. Buccilli, S. I. Cooper, C. Henderson, P. Rumerio, C. West, D. Arcaro, A. Avetisyan, T. Bose, D. Gastler, D. Rankin, C. Richardson, J. Rohlf, L. Sulak, D. Zou, G. Benelli, D. Cutts, A. Garabedian, J. Hakala, U. Heintz, J. M. Hogan, O. Jesus, K. H. M. Kwok, E. Laird, G. Landsberg, Z. Mao, M. Narain, S. Piperov, S. Sagir, E. Spencer, R. Syarif, R. Breedon, D. Burns, M. Calderon De La Barca Sanchez, S. Chauhan, M. Chertok, J. Conway, R. Conway, P. T. Cox, R. Erbacher, C. Flores, G. Funk, M. Gardner, W. Ko, R. Lander, C. Mclean, M. Mulhearn, D. Pellett, J. Pilot, S. Shalhout, M. Shi, J. Smith, M. Squires, D. Stolp, K. Tos, M. Tripathi, M. Bachtis, C. Bravo, R. Cousins, A. Dasgupta, A. Florent, J. Hauser, M. Ignatenko, N. Mccoll, D. Saltzberg, C. Schnaible, V. Valuev, M. Weber, E. Bouvier, K. Burt, R. Clare, J. Ellison, J. W. Gary, S. M. A. Ghiasi Shirazi, G. Hanson, J. Heilman, P. Jandir, E. Kennedy, F. Lacroix, O. R. Long, M. Olmedo Negrete, M. I. Paneva, A. Shrinivas, W. Si, H. Wei, S. Wimpenny, B. R. Yates, J. G. Branson, G. B. Cerati, S. Cittolin, M. Derdzinski, R. Gerosa, A. Holzner, D. Klein, V. Krutelyov, J. Letts, I. Macneill, D. Olivito, S. Padhi, M. Pieri, M. Sani, V. Sharma, S. Simon, M. Tadel, A. Vartak, S. Wasserbaech, C. Welke, J. Wood, F. Würthwein, A. Yagil, G. Zevi Della Porta, N. Amin, R. Bhandari, J. Bradmiller-Feld, C. Campagnari, A. Dishaw, V. Dutta, M. Franco Sevilla, C. George, F. Golf, L. Gouskos, J. Gran, R. Heller, J. Incandela, S. D. Mullin, A. Ovcharova, H. Qu, J. Richman, D. Stuart, I. Suarez, J. Yoo, D. Anderson, J. Bendavid, A. Bornheim, J. Bunn, J. Duarte, J. M. Lawhorn, A. Mott, H. B. Newman, C. Pena, M. Spiropulu, J. R. Vlimant, S. Xie, R. Y. Zhu, M. B. Andrews, T. Ferguson, M. Paulini, J. Russ, M. Sun, H. Vogel, I. Vorobiev, M. Weinberg, J. P. Cumalat, W. T. Ford, F. Jensen, A. Johnson, M. Krohn, S. Leontsinis, T. Mulholland, K. Stenson, S. R. Wagner, J. Alexander, J. Chaves, J. Chu, S. Dittmer, K. Mcdermott, N. Mirman, G. Nicolas Kaufman, J. R. Patterson, A. Rinkevicius, A. Ryd, L. Skinnari, L. Soffi, S. M. Tan, Z. Tao, J. Thom, J. Tucker, P. Wittich, M. Zientek, D. Winn, S. Abdullin, M. Albrow, G. Apollinari, A. Apresyan, S. Banerjee, L. A. T. Bauerdick, A. Beretvas, J. Berryhill, P. C. Bhat, G. Bolla, K. Burkett, J. N. Butler, H. W. K. Cheung, F. Chlebana, S. Cihangir, M. Cremonesi, V. D. Elvira, I. Fisk, J. Freeman, E. Gottschalk, L. Gray, D. Green, S. Grünendahl, O. Gutsche, D. Hare, R. M. Harris, S. Hasegawa, J. Hirschauer, Z. Hu, B. Jayatilaka, S. Jindariani, M. Johnson, U. Joshi, B. Klima, B. Kreis, S. Lammel, J. Linacre, D. Lincoln, R. Lipton, M. Liu, T. Liu, R. Lopes De Sá, J. Lykken, K. Maeshima, N. Magini, J. M. Marraffino, S. Maruyama, D. Mason, P. McBride, P. Merkel, S. Mrenna, S. Nahn, V. O’Dell, K. Pedro, O. Prokofyev, G. Rakness, L. Ristori, E. Sexton-Kennedy, A. Soha, W. J. Spalding, L. Spiegel, S. Stoynev, J. Strait, N. Strobbe, L. Taylor, S. Tkaczyk, N. V. Tran, L. Uplegger, E. W. Vaandering, C. Vernieri, M. Verzocchi, R. Vidal, M. Wang, H. A. Weber, A. Whitbeck, Y. Wu, D. Acosta, P. Avery, P. Bortignon, D. Bourilkov, A. Brinkerhoff, A. Carnes, M. Carver, D. Curry, S. Das, R. D. Field, I. K. Furic, J. Konigsberg, A. Korytov, J. F. Low, P. Ma, K. Matchev, H. Mei, G. Mitselmakher, D. Rank, L. Shchutska, D. Sperka, L. Thomas, J. Wang, S. Wang, J. Yelton, S. Linn, P. Markowitz, G. Martinez, J. L. Rodriguez, A. Ackert, T. Adams, A. Askew, S. Bein, S. Hagopian, V. Hagopian, K. F. Johnson, T. Kolberg, H. Prosper, A. Santra, R. Yohay, M. M. Baarmand, V. Bhopatkar, S. Colafranceschi, M. Hohlmann, D. Noonan, T. Roy, F. Yumiceva, M. R. Adams, L. Apanasevich, D. Berry, R. R. Betts, I. Bucinskaite, R. Cavanaugh, O. Evdokimov, L. Gauthier, C. E. Gerber, D. J. Hofman, K. Jung, I. D. Sandoval Gonzalez, N. Varelas, H. Wang, Z. Wu, M. Zakaria, J. Zhang, B. Bilki, W. Clarida, K. Dilsiz, S. Durgut, R. P. Gandrajula, M. Haytmyradov, V. Khristenko, J.-P. Merlo, H. Mermerkaya, A. Mestvirishvili, A. Moeller, J. Nachtman, H. Ogul, Y. Onel, F. Ozok, A. Penzo, C. Snyder, E. Tiras, J. Wetzel, K. Yi, B. Blumenfeld, A. Cocoros, N. Eminizer, D. Fehling, L. Feng, A. V. Gritsan, P. Maksimovic, J. Roskes, U. Sarica, M. Swartz, M. Xiao, C. You, A. Al-bataineh, P. Baringer, A. Bean, S. Boren, J. Bowen, J. Castle, L. Forthomme, R. P. Kenny, S. Khalil, A. Kropivnitskaya, D. Majumder, W. Mcbrayer, M. Murray, S. Sanders, R. Stringer, J. D. Tapia Takaki, Q. Wang, A. Ivanov, K. Kaadze, Y. Maravin, A. Mohammadi, L. K. Saini, N. Skhirtladze, S. Toda, F. Rebassoo, D. Wright, C. Anelli, A. Baden, O. Baron, A. Belloni, B. Calvert, S. C. Eno, C. Ferraioli, J. A. Gomez, N. J. Hadley, S. Jabeen, G. Y. Jeng, R. G. Kellogg, J. Kunkle, A. C. Mignerey, F. Ricci-Tam, Y. H. Shin, A. Skuja, M. B. Tonjes, S. C. Tonwar, D. Abercrombie, B. Allen, A. Apyan, V. Azzolini, R. Barbieri, A. Baty, R. Bi, K. Bierwagen, S. Brandt, W. Busza, I. A. Cali, M. D’Alfonso, Z. Demiragli, G. Gomez Ceballos, M. Goncharov, D. Hsu, Y. Iiyama, G. M. Innocenti, M. Klute, D. Kovalskyi, K. Krajczar, Y. S. Lai, Y.-J. Lee, A. Levin, P. D. Luckey, B. Maier, A. C. Marini, C. Mcginn, C. Mironov, S. Narayanan, X. Niu, C. Paus, C. Roland, G. Roland, J. Salfeld-Nebgen, G. S. F. Stephans, K. Tatar, D. Velicanu, J. Wang, T. W. Wang, B. Wyslouch, A. C. Benvenuti, R. M. Chatterjee, A. Evans, P. Hansen, S. Kalafut, S. C. Kao, Y. Kubota, Z. Lesko, J. Mans, S. Nourbakhsh, N. Ruckstuhl, R. Rusack, N. Tambe, J. Turkewitz, J. G. Acosta, S. Oliveros, E. Avdeeva, K. Bloom, D. R. Claes, C. Fangmeier, R. Gonzalez Suarez, R. Kamalieddin, I. Kravchenko, A. Malta Rodrigues, J. Monroy, J. E. Siado, G. R. Snow, B. Stieger, M. Alyari, J. Dolen, A. Godshalk, C. Harrington, I. Iashvili, J. Kaisen, D. Nguyen, A. Parker, S. Rappoccio, B. Roozbahani, G. Alverson, E. Barberis, A. Hortiangtham, A. Massironi, D. M. Morse, D. Nash, T. Orimoto, R. Teixeira De Lima, D. Trocino, R.-J. Wang, D. Wood, S. Bhattacharya, O. Charaf, K. A. Hahn, A. Kumar, N. Mucia, N. Odell, B. Pollack, M. H. Schmitt, K. Sung, M. Trovato, M. Velasco, N. Dev, M. Hildreth, K. Hurtado Anampa, C. Jessop, D. J. Karmgard, N. Kellams, K. Lannon, N. Marinelli, F. Meng, C. Mueller, Y. Musienko, M. Planer, A. Reinsvold, R. Ruchti, N. Rupprecht, G. Smith, S. Taroni, M. Wayne, M. Wolf, A. Woodard, J. Alimena, L. Antonelli, B. Bylsma, L. S. Durkin, S. Flowers, B. Francis, A. Hart, C. Hill, R. Hughes, W. Ji, B. Liu, W. Luo, D. Puigh, B. L. Winer, H. W. Wulsin, S. Cooperstein, O. Driga, P. Elmer, J. Hardenbrook, P. Hebda, D. Lange, J. Luo, D. Marlow, T. Medvedeva, K. Mei, I. Ojalvo, J. Olsen, C. Palmer, P. Piroué, D. Stickland, A. Svyatkovskiy, C. Tully, S. Malik, A. Barker, V. E. Barnes, S. Folgueras, L. Gutay, M. K. Jha, M. Jones, A. W. Jung, A. Khatiwada, D. H. Miller, N. Neumeister, J. F. Schulte, X. Shi, J. Sun, F. Wang, W. Xie, N. Parashar, J. Stupak, A. Adair, B. Akgun, Z. Chen, K. M. Ecklund, F. J. M. Geurts, M. Guilbaud, W. Li, B. Michlin, M. Northup, B. P. Padley, J. Roberts, J. Rorie, Z. Tu, J. Zabel, B. Betchart, A. Bodek, P. de Barbaro, R. Demina, Y. t. Duh, T. Ferbel, M. Galanti, A. Garcia-Bellido, J. Han, O. Hindrichs, A. Khukhunaishvili, K. H. Lo, P. Tan, M. Verzetti, A. Agapitos, J. P. Chou, Y. Gershtein, T. A. Gómez Espinosa, E. Halkiadakis, M. Heindl, E. Hughes, S. Kaplan, R. Kunnawalkam Elayavalli, S. Kyriacou, A. Lath, K. Nash, M. Osherson, H. Saka, S. Salur, S. Schnetzer, D. Sheffield, S. Somalwar, R. Stone, S. Thomas, P. Thomassen, M. Walker, A. G. Delannoy, M. Foerster, J. Heideman, G. Riley, K. Rose, S. Spanier, K. Thapa, O. Bouhali, A. Celik, M. Dalchenko, M. De Mattia, A. Delgado, S. Dildick, R. Eusebi, J. Gilmore, T. Huang, E. Juska, T. Kamon, R. Mueller, Y. Pakhotin, R. Patel, A. Perloff, L. Perniè, D. Rathjens, A. Safonov, A. Tatarinov, K. A. Ulmer, N. Akchurin, C. Cowden, J. Damgov, F. De Guio, C. Dragoiu, P. R. Dudero, J. Faulkner, E. Gurpinar, S. Kunori, K. Lamichhane, S. W. Lee, T. Libeiro, T. Peltola, S. Undleeb, I. Volobouev, Z. Wang, S. Greene, A. Gurrola, R. Janjam, W. Johns, C. Maguire, A. Melo, H. Ni, P. Sheldon, S. Tuo, J. Velkovska, Q. Xu, M. W. Arenton, P. Barria, B. Cox, J. Goodell, R. Hirosky, A. Ledovskoy, H. Li, C. Neu, T. Sinthuprasith, X. Sun, Y. Wang, E. Wolfe, F. Xia, C. Clarke, R. Harr, P. E. Karchin, J. Sturdy, D. A. Belknap, J. Buchanan, C. Caillol, S. Dasu, L. Dodd, S. Duric, B. Gomber, M. Grothe, M. Herndon, A. Hervé, P. Klabbers, A. Lanaro, A. Levine, K. Long, R. Loveless, T. Perry, G. A. Pierro, G. Polese, T. Ruggles, A. Savin, N. Smith, W. H. Smith, D. Taylor, N. Woods, CMS Collaboration

**Affiliations:** 1 Yerevan Physics Institute, Yerevan, Armenia; 2 Institut für Hochenergiephysik, Vienna, Austria; 3 Institute for Nuclear Problems, Minsk, Belarus; 4 National Centre for Particle and High Energy Physics, Minsk, Belarus; 5 Universiteit Antwerpen, Antwerpen, Belgium; 6 Vrije Universiteit Brussel, Brussels, Belgium; 7 Université Libre de Bruxelles, Brussels, Belgium; 8 Ghent University, Ghent, Belgium; 9 Université Catholique de Louvain, Louvain-la-Neuve, Belgium; 10 Université de Mons, Mons, Belgium; 11 Centro Brasileiro de Pesquisas Fisicas, Rio de Janeiro, Brazil; 12 Universidade do Estado do Rio de Janeiro, Rio de Janeiro, Brazil; 13 Universidade Estadual Paulista , Universidade Federal do ABC, São Paulo, Brazil; 14 Institute for Nuclear Research and Nuclear Energy, Sofia, Bulgaria; 15 University of Sofia, Sofia, Bulgaria; 16 Beihang University, Beijing, China; 17 Institute of High Energy Physics, Beijing, China; 18 State Key Laboratory of Nuclear Physics and Technology, Peking University, Beijing, China; 19 Universidad de Los Andes, Bogotá, Colombia; 20 Faculty of Electrical Engineering Mechanical Engineering and Naval Architecture, University of Split, Split, Croatia; 21 Faculty of Science, University of Split, Split, Croatia; 22 Institute Rudjer Boskovic, Zagreb, Croatia; 23 University of Cyprus, Nicosia, Cyprus; 24 Charles University, Prague, Czech Republic; 25 Universidad San Francisco de Quito, Quito, Ecuador; 26 Academy of Scientific Research and Technology of the Arab Republic of Egypt, Egyptian Network of High Energy Physics, Cairo, Egypt; 27 National Institute of Chemical Physics and Biophysics, Tallinn, Estonia; 28 Department of Physics, University of Helsinki, Helsinki, Finland; 29 Helsinki Institute of Physics, Helsinki, Finland; 30 Lappeenranta University of Technology, Lappeenranta, Finland; 31 IRFU, CEA, Université Paris-Saclay, Gif-sur-Yvette, France; 32 Laboratoire Leprince-Ringuet, Ecole Polytechnique, IN2P3-CNRS, Palaiseau, France; 33 Institut Pluridisciplinaire Hubert Curien (IPHC), Université de Strasbourg, CNRS-IN2P3, Strasbourg, France; 34 Centre de Calcul de l’Institut National de Physique Nucleaire et de Physique des Particules, CNRS/IN2P3, Villeurbanne, France; 35 Université de Lyon, Université Claude Bernard Lyon 1, CNRS-IN2P3, Institut de Physique Nucléaire de Lyon, Villeurbanne, France; 36 Georgian Technical University, Tbilisi, Georgia; 37 Tbilisi State University, Tbilisi, Georgia; 38 RWTH Aachen University, I. Physikalisches Institut, Aachen, Germany; 39 RWTH Aachen University, III. Physikalisches Institut A, Aachen, Germany; 40 RWTH Aachen University, III. Physikalisches Institut B, Aachen, Germany; 41 Deutsches Elektronen-Synchrotron, Hamburg, Germany; 42 University of Hamburg, Hamburg, Germany; 43 Institut für Experimentelle Kernphysik, Karlsruhe, Germany; 44 Institute of Nuclear and Particle Physics (INPP), NCSR Demokritos, Aghia Paraskevi, Greece; 45 National and Kapodistrian University of Athens, Athens, Greece; 46 University of Ioánnina, Ioánnina, Greece; 47 MTA-ELTE Lendület CMS Particle and Nuclear Physics Group, Eötvös Loránd University, Budapest, Hungary; 48 Wigner Research Centre for Physics, Budapest, Hungary; 49 Institute of Nuclear Research ATOMKI, Debrecen, Hungary; 50 Institute of Physics, University of Debrecen, Debrecen, Hungary; 51 Indian Institute of Science (IISc), Bangalore, India; 52 National Institute of Science Education and Research, Bhubaneswar, India; 53 Panjab University, Chandigarh, India; 54 University of Delhi, Delhi, India; 55 Saha Institute of Nuclear Physics, Kolkata, India; 56 Indian Institute of Technology Madras, Madras, India; 57 Bhabha Atomic Research Centre, Mumbai, India; 58 Tata Institute of Fundamental Research-A, Mumbai, India; 59 Tata Institute of Fundamental Research-B, Mumbai, India; 60 Indian Institute of Science Education and Research (IISER), Pune, India; 61 Institute for Research in Fundamental Sciences (IPM), Tehran, Iran; 62 University College Dublin, Dublin, Ireland; 63 INFN Sezione di Bari , Università di Bari , Politecnico di Bari, Bari, Italy; 64 INFN Sezione di Bologna , Università di Bologna, Bologna, Italy; 65 INFN Sezione di Catania , Università di Catania, Catania, Italy; 66 INFN Sezione di Firenze , Università di Firenze, Firenze, Italy; 67 INFN Laboratori Nazionali di Frascati, Frascati, Italy; 68 INFN Sezione di Genova, Università di Genova, Genoa, Italy; 69 INFN Sezione di Milano-Bicocca, Università di Milano-Bicocca, Milan, Italy; 70 INFN Sezione di Napoli , Università di Napoli ‘Federico II’ , Napoli, Italy, Università della Basilicata , Potenza, Italy, Università G. Marconi, Rome, Italy; 71 INFN Sezione di Padova , Università di Padova , Padova, Italy, Università di Trento, Trento, Italy; 72 INFN Sezione di Pavia , Università di Pavia, Pavia, Italy; 73 INFN Sezione di Perugia , Università di Perugia, Perugia, Italy; 74 INFN Sezione di Pisa , Università di Pisa , Scuola Normale Superiore di Pisa, Pisa, Italy; 75 INFN Sezione di Roma , Università di Roma, Rome, Italy; 76 INFN Sezione di Torino , Università di Torino , Torino, Italy, Università del Piemonte Orientale, Novara, Italy; 77 INFN Sezione di Trieste , Università di Trieste, Trieste, Italy; 78 Kyungpook National University, Daegu, Korea; 79 Chonbuk National University, Jeonju, Korea; 80 Chonnam National University, Institute for Universe and Elementary Particles, Kwangju, Korea; 81 Hanyang University, Seoul, Korea; 82 Korea University, Seoul, Korea; 83 Seoul National University, Seoul, Korea; 84 University of Seoul, Seoul, Korea; 85 Sungkyunkwan University, Suwon, Korea; 86 Vilnius University, Vilnius, Lithuania; 87 National Centre for Particle Physics, Universiti Malaya, Kuala Lumpur, Malaysia; 88 Centro de Investigacion y de Estudios Avanzados del IPN, Mexico City, Mexico; 89 Universidad Iberoamericana, Mexico City, Mexico; 90 Benemerita Universidad Autonoma de Puebla, Puebla, Mexico; 91 Universidad Autónoma de San Luis Potosí, San Luis Potosí, Mexico; 92 University of Auckland, Auckland, New Zealand; 93 University of Canterbury, Christchurch, New Zealand; 94 National Centre for Physics, Quaid-I-Azam University, Islamabad, Pakistan; 95 National Centre for Nuclear Research, Swierk, Poland; 96 Institute of Experimental Physics, Faculty of Physics, University of Warsaw, Warsaw, Poland; 97 Laboratório de Instrumentação e Física Experimental de Partículas, Lisbon, Portugal; 98 Joint Institute for Nuclear Research, Dubna, Russia; 99 Petersburg Nuclear Physics Institute, Gatchina (St. Petersburg), Russia; 100 Institute for Nuclear Research, Moscow, Russia; 101 Institute for Theoretical and Experimental Physics, Moscow, Russia; 102 Moscow Institute of Physics and Technology, Moscow, Russia; 103 National Research Nuclear University ’Moscow Engineering Physics Institute’ (MEPhI), Moscow, Russia; 104 P.N. Lebedev Physical Institute, Moscow, Russia; 105 Skobeltsyn Institute of Nuclear Physics, Lomonosov Moscow State University, Moscow, Russia; 106 Novosibirsk State University (NSU), Novosibirsk, Russia; 107 State Research Center of Russian Federation, Institute for High Energy Physics, Protvino, Russia; 108 University of Belgrade, Faculty of Physics and Vinca Institute of Nuclear Sciences, Belgrade, Serbia; 109 Centro de Investigaciones Energéticas Medioambientales y Tecnológicas (CIEMAT), Madrid, Spain; 110 Universidad Autónoma de Madrid, Madrid, Spain; 111 Universidad de Oviedo, Oviedo, Spain; 112 Instituto de Física de Cantabria (IFCA), CSIC-Universidad de Cantabria, Santander, Spain; 113 CERN, European Organization for Nuclear Research, Geneva, Switzerland; 114 Paul Scherrer Institut, Villigen, Switzerland; 115 Institute for Particle Physics, ETH Zurich, Zurich, Switzerland; 116 Universität Zürich, Zurich, Switzerland; 117 National Central University, Chung-Li, Taiwan; 118 National Taiwan University (NTU), Taipei, Taiwan; 119 Chulalongkorn University, Faculty of Science, Department of Physics, Bangkok, Thailand; 120 Cukurova University-Physics Department, Science and Art Faculty, Adana, Turkey; 121 Middle East Technical University, Physics Department, Ankara, Turkey; 122 Bogazici University, Istanbul, Turkey; 123 Istanbul Technical University, Istanbul, Turkey; 124 Institute for Scintillation Materials of National Academy of Science of Ukraine, Kharkov, Ukraine; 125 National Scientific Center, Kharkov Institute of Physics and Technology, Kharkov, Ukraine; 126 University of Bristol, Bristol, UK; 127 Rutherford Appleton Laboratory, Didcot, UK; 128 Imperial College, London, UK; 129 Brunel University, Uxbridge, UK; 130 Baylor University, Waco, USA; 131 Catholic University of America, Washington, DC, USA; 132 The University of Alabama, Tuscaloosa, USA; 133 Boston University, Boston, USA; 134 Brown University, Providence, USA; 135 University of California, Davis, Davis, USA; 136 University of California, Los Angeles, USA; 137 University of California, Riverside, Riverside, USA; 138 University of California, San Diego, La Jolla, USA; 139 University of California, Santa Barbara-Department of Physics, Santa Barbara, USA; 140 California Institute of Technology, Pasadena, USA; 141 Carnegie Mellon University, Pittsburgh, USA; 142 University of Colorado Boulder, Boulder, USA; 143 Cornell University, Ithaca, USA; 144 Fairfield University, Fairfield, USA; 145 Fermi National Accelerator Laboratory, Batavia, USA; 146 University of Florida, Gainesville, USA; 147 Florida International University, Miami, USA; 148 Florida State University, Tallahassee, USA; 149 Florida Institute of Technology, Melbourne, USA; 150 University of Illinois at Chicago (UIC), Chicago, USA; 151 The University of Iowa, Iowa City, USA; 152 Johns Hopkins University, Baltimore, USA; 153 The University of Kansas, Lawrence, USA; 154 Kansas State University, Manhattan, USA; 155 Lawrence Livermore National Laboratory, Livermore, USA; 156 University of Maryland, College Park, USA; 157 Massachusetts Institute of Technology, Cambridge, USA; 158 University of Minnesota, Minneapolis, USA; 159 University of Mississippi, Oxford, USA; 160 University of Nebraska-Lincoln, Lincoln, USA; 161 State University of New York at Buffalo, Buffalo, USA; 162 Northeastern University, Boston, USA; 163 Northwestern University, Evanston, USA; 164 University of Notre Dame, Notre Dame, USA; 165 The Ohio State University, Columbus, USA; 166 Princeton University, Princeton, USA; 167 University of Puerto Rico, Mayaguez, USA; 168 Purdue University, West Lafayette, USA; 169 Purdue University Northwest, Hammond, USA; 170 Rice University, Houston, USA; 171 University of Rochester, Rochester, USA; 172 Rutgers, The State University of New Jersey, Piscataway, USA; 173 University of Tennessee, Knoxville, USA; 174 Texas A&M University, College Station, USA; 175 Texas Tech University, Lubbock, USA; 176 Vanderbilt University, Nashville, USA; 177 University of Virginia, Charlottesville, USA; 178 Wayne State University, Detroit, USA; 179 University of Wisconsin-Madison, Madison, WI USA; 180 CERN, 1211 Geneva 23, Switzerland

## Abstract

A measurement of the top quark mass is reported in events containing a single top quark produced via the electroweak *t* channel. The analysis is performed using data from proton-proton collisions collected with the CMS detector at the LHC at a centre-of-mass energy of 8 TeV, corresponding to an integrated luminosity of 19.7 fb$^{-1}$. Top quark candidates are reconstructed from their decay to a $\mathrm {W}$ boson and a b quark, with the $\mathrm {W}$ boson decaying leptonically to a muon and a neutrino. The final state signature and kinematic properties of single top quark events in the *t* channel are used to enhance the purity of the sample, suppressing the contribution from top quark pair production. A fit to the invariant mass distribution of reconstructed top quark candidates yields a value of the top quark mass of $172.95 \pm 0.77\,\text {(stat)} ^{+0.97}_{-0.93}\,\text {(syst)} \,\text {GeV}$. This result is in agreement with the current world average, and represents the first measurement of the top quark mass in event topologies not dominated by top quark pair production, therefore contributing to future averages with partially uncorrelated systematic uncertainties and a largely uncorrelated statistical uncertainty.

## Introduction

All previously published measurements of the top quark mass have been obtained using samples of top quark-antiquark pairs. A combination of measurements from the CDF and D0 experiments at the Tevatron and ATLAS and CMS experiments at the LHC yields a value of $173.34 \pm 0.27\,\text {(stat)} \pm 0.71\,\text {(syst)} \,\text {GeV}$ for the top quark mass $m_{\mathrm{t}}$ [1]. Measuring $m_{\mathrm{t}}$ in single top quark production enriches the range of available measurements, exploiting a sample which is almost statistically independent from those used by previous ones, and with systematic uncertainties partially uncorrelated from those considered in $\mathrm{t}\overline{\mathrm{t}}$ production. Because of the different production mechanism, the mass extraction is affected differently by the modelling of both perturbative effects, such as initial- and final-state radiation, and nonperturbative effects, such as colour reconnection, in quantum chromodynamics (QCD). Some discussion on these topics, though mainly restricted to the case of pair production, can be found in Refs. [2, 3]. In perspective, the lower level of gluon radiation and final state combinatorial arrangements with respect to $\mathrm{t}\overline{\mathrm{t}}$ production will make this channel a good candidate for precision measurements of $m_{\mathrm{t}}$ when larger samples of events are available.

At the CERN LHC, top quarks are mainly produced as $\mathrm{t}\overline{\mathrm{t}}$ pairs, through gluon-gluon fusion or quark-antiquark annihilation, mediated by the strong interaction. The standard model (SM) predicts single top quark production through electroweak processes, with a rate about one third that of the $\mathrm{t}\overline{\mathrm{t}}$ production cross section. This has been confirmed by observations at the Tevatron  [4] and LHC  [5, 6].

In this paper, top quark candidates are reconstructed via their decay to a $\mathrm {W}$ boson and a $\mathrm{b}$ quark, with the $\mathrm {W}$ boson decaying to a muon and a neutrino. The event selection is tailored, before looking at data in the signal region, to enhance the single top quark content in the final sample and so have a result as independent as possible from those obtained using $\mathrm{t}\overline{\mathrm{t}}$ events.

The paper is organised as follows. Section 2 describes the CMS detector, followed by information about the data sample and simulation used in the analysis in Sect. 3. The selection of events and the reconstruction of the top quark candidates is given in Sect. 4, and the description of the maximum-likelihood fit to derive the top quark mass is in Sect. 5. Section 6 describes the systematic uncertainties affecting the measurement and Sect. 7 summarises the results.

## The CMS detector

The central feature of the CMS apparatus is a superconducting solenoid of 6 $\text {\,m}$ internal diameter, providing a magnetic field of 3.8 $\text {\,T}$. Within the solenoid volume are a silicon pixel and strip tracker, a lead tungstate crystal electromagnetic calorimeter, and a brass and scintillator hadron calorimeter, each composed of a barrel and two endcap sections. Forward calorimeters extend the pseudorapidity coverage provided by the barrel and endcap detectors. Muons are measured in gas-ionisation detectors embedded in the steel flux-return yoke outside the solenoid.

A more detailed description of the CMS detector, together with a definition of the coordinate system used and the relevant kinematic variables, can be found in Ref. [7].

## Data and simulated samples

The measurement reported here is performed using the $\sqrt{s}=$8 TeV proton-proton collision data sample collected in 2012 with the CMS detector, corresponding to an integrated luminosity of 19.7 fb$^{-1}$.

**Fig. 1:**
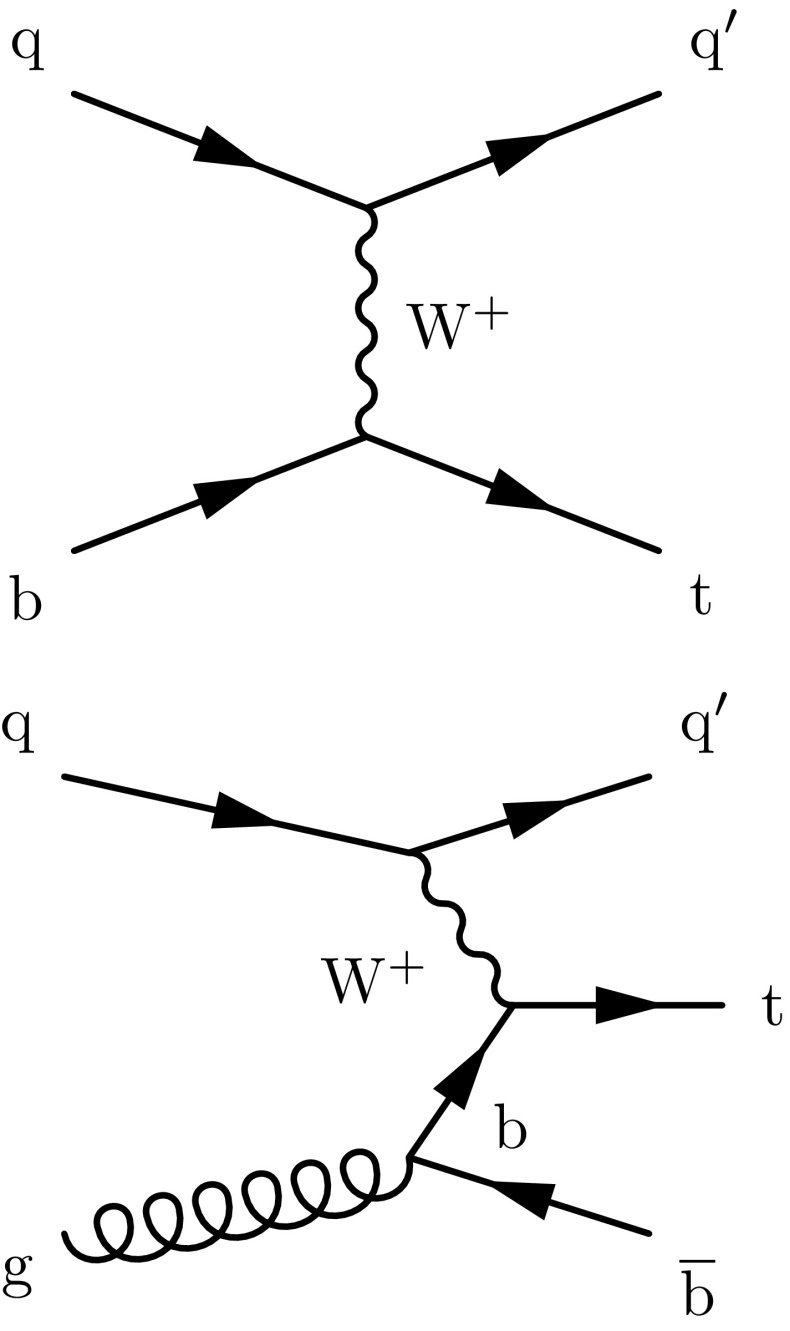
Feynman diagrams representing the dominant single top quark production mechanisms in the *t* channel

At the lowest order in perturbation theory, single top quark production proceeds through the *t*-channel, *s*-channel, and associated $\mathrm{t} \mathrm {W}$ production modes. The *t* channel provides the largest contribution to the single top quark cross section. The corresponding amplitude can be calculated using one of two different schemes [8–10]: in the 5-flavour scheme, b quarks are considered as coming from the interacting proton, and the leading-order (LO) diagram is a $2 \rightarrow 2$ process (Fig. 1, upper); in the 4-flavour scheme, b quarks are not present in the initial state, and the LO diagram is a $2 \rightarrow 3$ process (Fig. 1, lower). The predicted *t*-channel single top quark cross section for pp collisions at a centre-of-mass energy of 8 TeV is $\sigma _{\mathrm{t}}= 54.9^{+2.3}_{-1.9}$ $\text {\,pb}$ for the top quark and $\sigma _{\overline{\mathrm{t}}}=29.7^{+1.7}_{-1.5}$ $\text {\,pb}$ for the top antiquark. These values are obtained by a next-to-leading-order (NLO) calculation in quantum QCD with hathor v.2.1 [11, 12], assuming a top quark mass of 172.5$\,\text {GeV}$. The parton distribution functions (PDFs) and $\alpha _S$ uncertainties are calculated using the PDF4LHC prescription [13, 14] with the MSTW2008 68% confidence level (CL) NLO [15, 16], CT10 NLO [17], and NNPDF2.3 [18] PDF sets.

At 8 TeV, the predicted $\mathrm{t}\overline{\mathrm{t}}$ production cross section is $\sigma (\mathrm{t}\overline{\mathrm{t}}) = 252.9^{+6.4}_{-8.6}\,\text { (scale)} \pm 11.7\,(\mathrm {PDF} + \alpha _S)\,\text {\,pb}$ as calculated with the Top++2.0 program to next-to-next-to-leading order in perturbative QCD, including soft-gluon resummation to next-to-next-to-leading-log order (see Ref. [19] and references therein), and assuming a top quark mass of 172.5$\,\text {GeV}$. In this calculation, the total scale uncertainty is obtained from the independent variation of the factorisation and renormalisation scales, $\mu _\mathrm {F}$ and $\mu _\mathrm {R}$, by a factor 2 and 1/2; the total PDF and $\alpha _S$ uncertainties are estimated following the PDF4LHC prescription [14] with the MSTW2008 68% CL NNLO [16], CT10 NNLO [18], and NNPDF2.3 [20] FFN PDF sets.

Simulated events are used to optimise the event selection and to study the background processes and the expected performance of the analysis. The signal *t*-channel events are generated with the powheg generator, version 1.0 [21], in the 5-flavour scheme, interfaced with pythia  [22], version 6.426, for parton showering and hadronisation. Single top quark *s*-channel and tW associated production are considered as backgrounds for this measurement and simulated with the same generator. Top quark pair production, single vector boson production associated with jets (referred to as $\mathrm {W}$/${\mathrm{Z}}$ +jets in the following), and double vector boson (diboson) production are amongst the background processes taken into consideration and have been simulated with the MadGraph generator, version 5.148 [23], interfaced with pythia for parton showering. The pythia generator is used to simulate QCD multijet event samples enriched with isolated muons. The value of the top quark mass used in all simulated samples is 172.5$\,\text {GeV}$. All samples are generated using the CTEQ6.6M [24] PDF set and use the Z2* underlying event tune [25]. The factorisation and renormalisation scales are both set to $m_{\mathrm {t}}$ for the single top quark samples, while a dynamic scale is used for the other samples, defined as the sum in quadrature of the transverse momentum ($p_{\mathrm {T}}$) and the mass of the particles produced in the central process. The passage of particles through the detector is simulated using the Geant4 toolkit [26]. The simulation includes additional overlapping pp collisions (pileup) with a multiplicity that is tuned to match the one observed in data.

## Event selection and reconstruction

Signal events are characterised by a single isolated muon, momentum imbalance due to the presence of a neutrino, and one central b jet from the top quark decay. In addition, events often feature the presence of a light quark jet in the forward direction, from the hard-scattering process.

The online selection requires the presence of one isolated muon candidate with $p_{\mathrm {T}}$ greater than 24$\,\text {GeV}$ and absolute value of the pseudorapidity ($\eta$) below 2.1. Events are required to have at least one primary vertex reconstructed from at least four tracks, with a distance from the nominal beam-interaction point of less than 24 cm along the *z* axis and less than 2 cm in the transverse plane. In cases where more than one primary vertex is found, the one featuring the largest value of $\Sigma p_{\mathrm {T}} ^2$ is retained (“leading vertex”), where the sum runs over all the tracks assigned to that vertex.

All particles are reconstructed and identified with the CMS particle-flow algorithm [27, 28]. Muon candidates are further required to have $p_{\mathrm {T}} > 26\,\text {GeV}$, thus ensuring they are selected in the region of maximal trigger efficiency. Muon candidates are also required to be isolated. This is ensured by requiring $I_{\text {rel}} < 0.12$, where $I_{\text {rel}}$ is defined as the sum of the transverse energies deposited by long-lived charged hadrons, photons, and neutral hadrons in a cone of size $\Delta R =\sqrt{\smash [b]{( \Delta \eta )^2 + ( \Delta \phi )^2}} = 0.4$ around the muon direction ($\phi$ being the azimuthal angle, in radians), divided by the muon $p_{\mathrm {T}}$ itself. An offset correction is applied to remove the additional energy included in the jets that come from pileup [29]. Events are rejected if an additional muon (electron) candidate is present, passing the selection criteria $p_{\mathrm {T}} > 10\, (20)$ $\,\text {GeV}$, $|\eta |< 2.5$, and $I_{\text {rel}} < 0.2\ (0.15)$.

To define jets, the reconstructed particles are clustered using the anti-$k_{\mathrm {T}}$ algorithm [30] with a distance parameter of 0.5. Charged particles are excluded if they originate from a primary vertex that is not the leading vertex. The energy deposition in the jet due to neutral pileup particles is inferred and subtracted by considering charged pileup particles inside the jet cone. Additional corrections to the jet energies are derived from the study of dijet events and photon+jets events [31]. Jets are required to have $|\eta | < 4.7$ and to have a corrected transverse energy greater than 40$\,\text {GeV}$. Jets associated with the hadronisation of b quarks (“b jets”) are identified using a $\mathrm{b}$ tagging algorithm based on the 3D impact parameter of the tracks in the jet to define a $\mathrm{b}$ tagging discriminator [32]. The threshold for this variable is chosen such that the probability to misidentify jets coming from the hadronisation of light quarks ($\mathrm{u}$, $\mathrm{d}$, $\mathrm{s}$) or gluons is small (0.1%), while ensuring an efficiency of 46% for selecting jets coming from b quarks, as determined from the simulation of events with top quark topologies. Event weights are applied to adjust the b jet yields in the simulation to account for differences in the $\mathrm{b}$ tagging efficiency between data and simulation.

The missing transverse momentum (${\vec p}_{\mathrm {T}}^{\text {miss}}$) is calculated as the negative vector sum of the transverse momenta of all reconstructed particles. Corrections to the jet energies, as well as an offset correction accounting for pileup interactions, are propagated to ${\vec p}_{\mathrm {T}}^{\text {miss}}$. The missing transverse momentum magnitude ($p_{\mathrm {T}} ^\text {miss}$) is required to exceed 50$\,\text {GeV}$, to suppress the QCD multijet background.

To reject jets from pileup, non $\mathrm{b}$-tagged jets are rejected if the root-mean-square $\eta$-$\phi$ radius of the particles constituting the jet with respect to the jet axis is larger than 0.025. To suppress background from QCD multijet events, the transverse mass of the $\mathrm {W}$ boson $m_\mathrm {T}(\mathrm {W})$ must be larger than 50$\,\text {GeV}$, where $m_\mathrm {T}(\mathrm {W})$ is constructed from the missing transverse momentum and muon transverse momentum vectors as

1 $$\begin{aligned} m_{\mathrm {T}}(\mathrm {W}) = \sqrt{\left( p_{\mathrm {T}} ^{\mu } + p_{\mathrm {T}} ^\text {miss} \right) ^2 - \left( p_x^{\mu } + p^{\text {miss}}_{\mathrm {T},x} \right) ^2 - \left( p_y^{\mu } + p^{\text {miss}}_{\mathrm {T},y} \right) ^2}. \end{aligned}$$

The same event reconstruction and selection of top quark candidates adopted by the CMS single top quark *t*-channel cross section measurement at 8 TeV in Ref. [5] is used. Due to the detector acceptance and jet selection requirements, most signal events are characterised by the presence of two reconstructed jets, one of which comes from the hadronisation of a $\mathrm{b}$ quark. Therefore, events with two reconstructed jets, exactly one of which is b tagged, constitute the “signal region” (referred to as ‘2J1T’ in the following). Other event topologies are used to study background properties: the sample with two reconstructed jets, neither of which is b tagged (‘2J0T’) is dominated by $\mathrm {W}$+jets events; the sample with three reconstructed jets, where two jets are b tagged (‘3J2T’) is dominated by $\mathrm{t}\overline{\mathrm{t}}$ events. For all topologies considered, the jet with the highest value of the $\mathrm{b}$ tagging discriminator is used for top quark reconstruction, while that with the lowest value is taken to be the light-quark jet associated with top quark production (Fig. 1).

To enrich the sample in single top quark events, further requirements are applied to variables that exhibit good discriminating power with respect to $\mathrm{t}\overline{\mathrm{t}}$ events, as described below. The selection criteria have been chosen after studying their effect on the purity of the sample, while verifying that the statistical uncertainty achievable on the top quark mass would not be excessively degraded.

**Fig. 2:**
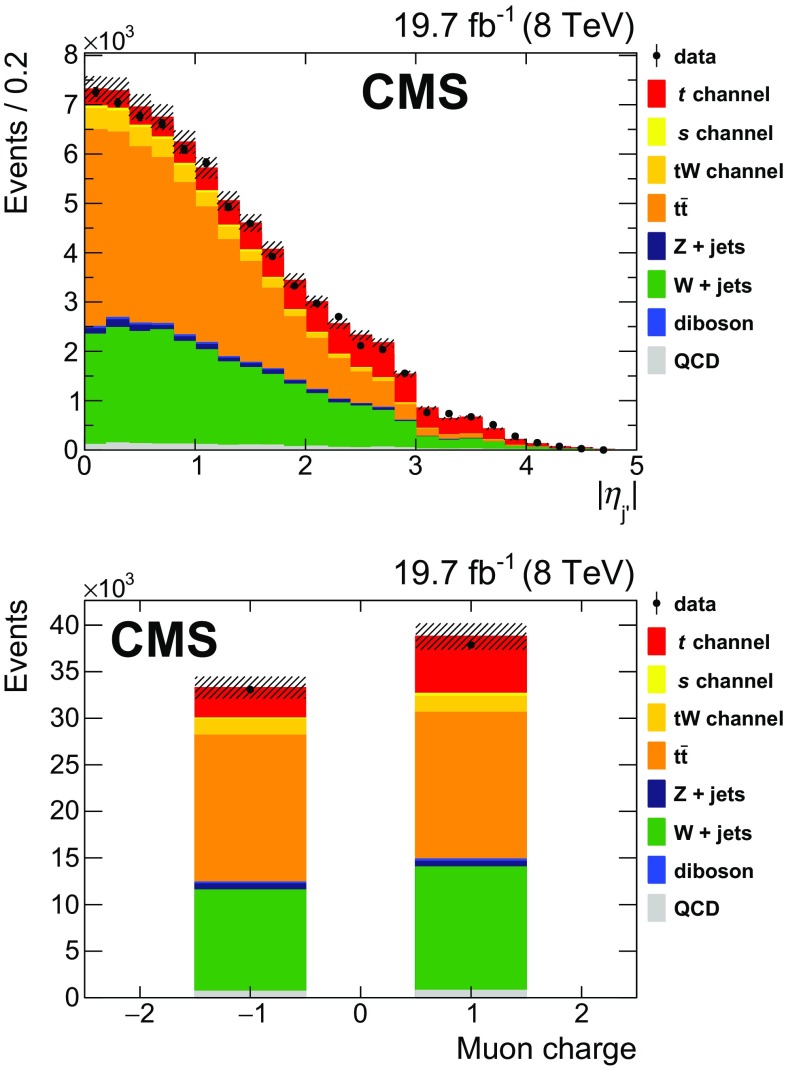
Distribution of the light-quark jet pseudorapidity (*upper*) and of the muon charge (*lower*) for all top quark candidates in the muonic decay channel. *Points with error bars* represent data, *stacked histograms* show expected contributions from Monte Carlo simulation. The *hatched area* represents the uncertainty on the Monte Carlo predictions associated to the finite size of the samples and their normalization, and the integrated luminosity

A feature of single top quark production in the *t* channel is that the top quark is accompanied by a light-quark jet (the quark labelled $\mathrm{q} '$ in Fig. 1), which is produced in a more forward direction than jets coming from $\mathrm{t}\overline{\mathrm{t}}$ production or other background processes. This is reflected in the distribution of the absolute value of the pseudorapidity of the light-quark jet $|\eta _{\mathrm {j}'} |$, shown in Fig. 2 (upper) for all reconstructed top quark candidates. A requirement of $|\eta _{\mathrm {j}'} | > 2.5$ is applied to the sample. The stability of the selection has been checked by verifying that, if the events with $|\eta _{\mathrm {j}'} | > 4$ were excluded, the final result would not be affected.

In *t*-channel single top quark production, top quarks are produced more frequently than top antiquarks due to the charge asymmetry of the proton-proton initial state [33], as seen in the muon charge distribution (Fig. 2, lower). To obtain as pure a sample as possible, only events with positively charged muons are retained.

## Determination of the top quark mass

For each selected event, the top quark mass is reconstructed from the invariant mass $m_{\mu \nu \mathrm{b}}$ calculated from the muon, the neutrino, and the $\mathrm{b}$ jet. The 4-momenta of the muon and the jet are measured, while, for the neutrino, the 4-momentum is determined by using the missing transverse momentum in the event and a kinematical constraint on the $\mathrm {\mu }$ $\nu$ invariant mass, required to be consistent with the mass $m_{\mathrm {W}}$ of the $\mathrm {W}$ boson [34]:

2 $$\begin{aligned} m_{\mathrm {W}}^2= & {} \left( E^{\mu } + \sqrt{(p_{\mathrm {T}} ^\text {miss})^2+(p^{\nu }_{z})^2} \right) ^2 - \left( p_x^{\mu } + p^{\text {miss}}_{\mathrm {T},x} \right) ^2 \nonumber \\&- \left( p_y^{\mu } + p^{\text {miss}}_{\mathrm {T},y} \right) ^2-\left( p_{z}^{\mu } + p_{z}^{\nu } \right) ^2 , \end{aligned}$$

where $E^{\mu }$ is the muon energy, $p_{x}^{\mu }$, $p_{y}^{\mu }$ and $p_{z}^{\mu }$ are the components of the muon momentum, $p_{z}^{\nu }$ is the longitudinal component of the neutrino momentum, and $p_{\mathrm {T}} ^\text {miss}$ is used for the transverse components of the neutrino momentum. Equation 2 is quadratic in $p_{z}^{\nu }$: when two real solutions are found, the one with the smallest value of $|p_{z}^{\nu }|$ is taken; in the case of complex solutions, the imaginary component is eliminated by modifying $p^{\text {miss}}_{\mathrm {T},x}$ and $p^{\text {miss}}_{\mathrm {T},y}$ independently, so as to give $m_\mathrm {T}(\mathrm {W}) = m_{\mathrm {W}}$  [35].

**Fig. 3:**
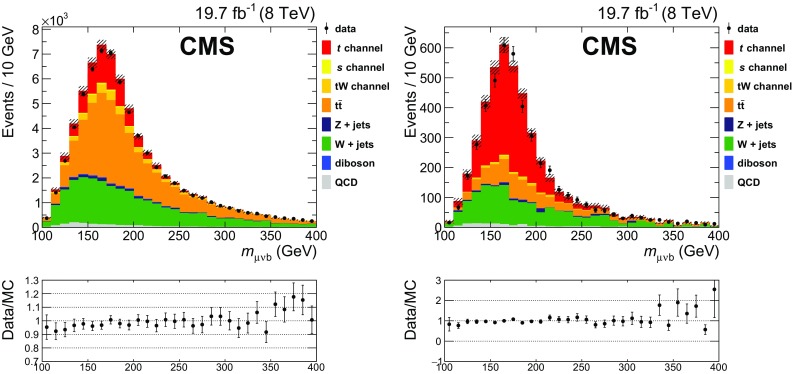
Reconstructed $\mu \nu \mathrm{b}$ invariant mass distribution for data (*points with error bars*) and Monte Carlo events (*stacked histograms*). (*Left*) initial selection; (*right*) final selection after the charge and light-quark jet pseudorapidity requirements. The ratio of the observed number of events in data to the number predicted by simulation is shown in the *lower plots*. The *hatched area* represents the uncertainty on the Monte Carlo predictions associated to the finite size of the samples and their normalization, and the integrated luminosity

Figure 3 shows the $m_{\mu \nu \mathrm{b}}$ distributions before and after the final event selection. According to Monte Carlo simulation, after the final selection, 73% of the reconstructed top quarks come from single top quark production, and of these about 97% come from *t*-channel production.

The top quark mass is measured with an extended unbinned maximum-likelihood fit to the $m_{\mu \nu \mathrm{b}}$ distribution. The numbers of events for the various contributions, except for the single top quark *t*-channel one, are fixed to the values extracted from simulation, taking into account the different theoretical cross sections [5]. The description of the parameterisation of the signal and background components used in the fit is presented below. The free parameters of the fit are the number of single top quark signal events and the parameters of the signal shape.

### Parameterisation of top quark components

The shapes of the $m_{\mu \nu \mathrm{b}}$ distributions for samples where a top quark is present are studied using simulated events.

**Fig. 4:**
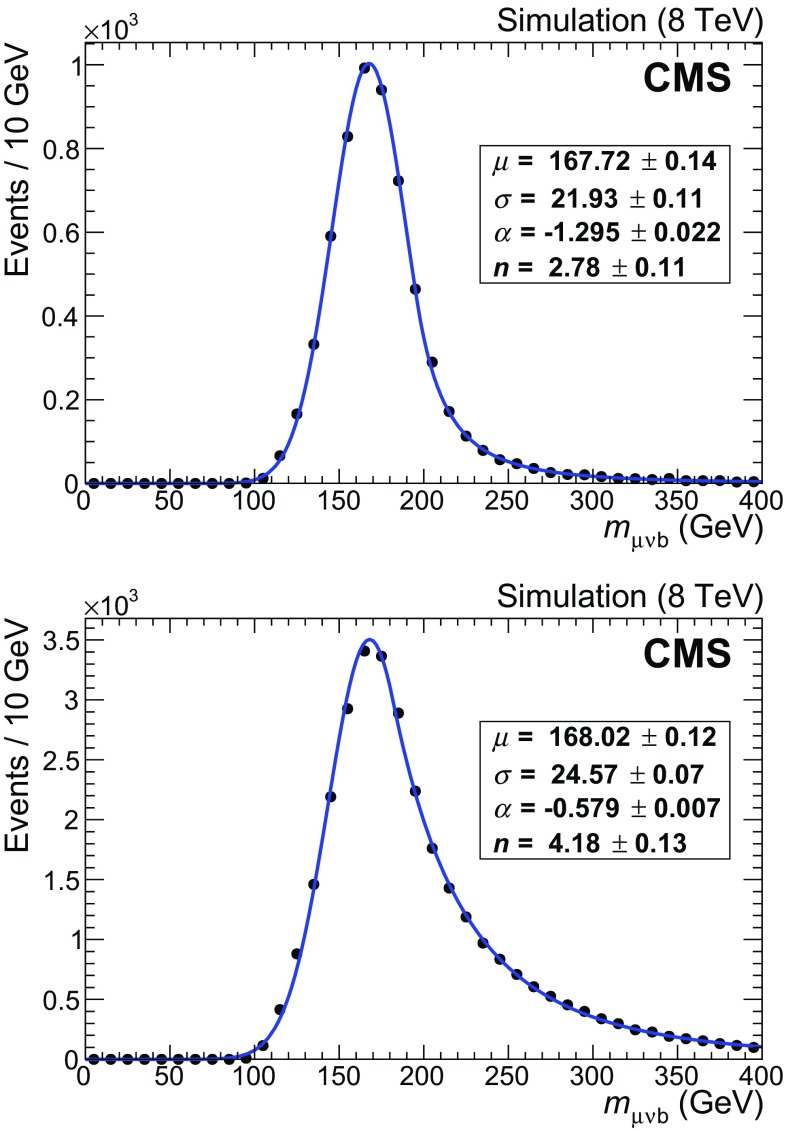
Reconstructed $\mu \nu \mathrm{b}$ invariant mass from Monte Carlo simulated events for single top quark *t* channel (*upper*) and $\mathrm{t}\overline{\mathrm{t}}$ (*lower*). The *continuous lines* show the results of fits to Crystal Ball shapes

The $\mathrm{t}\overline{\mathrm{t}}$ component exhibits a wider peak, with a larger high-mass tail, compared to the single top quark *t*-channel component. The simulation shows that the number of muon and $\mathrm{b}$ jet pairs correctly assigned to the parent top quark is around 55% for $\mathrm{t}\overline{\mathrm{t}}$ events, while this fraction exceeds 90% for signal events. Both contributions can be fitted by Crystal Ball functions [36], with independent parameters $\mu$ and $\sigma$ representing the Gaussian core, and $\alpha$ and *n* describing where the power-law tail begins and the exponent of the tail, respectively. The distributions obtained from the simulated samples before the final selection are shown in Fig. 4. The difference between the values of the $\mu$ parameter of the Crystal Ball function obtained from the fits is $m_{\mathrm{t}}(\text {t channel}) - m_{\mathrm{t}}(\mathrm{t}\overline{\mathrm{t}}) = 0.30 \pm 0.17\,\text {GeV}$, where the uncertainty is the statistical uncertainty from the fit.

The remaining single top quark components (*s*-channel and $\mathrm{t} \mathrm {W}$ production) account for only about 3.5% of the final sample and their contribution is absorbed in the $\mathrm{t}\overline{\mathrm{t}}$ component, since their distributions exhibit broader peaks with respect to the *t* channel.

**Fig. 5:**
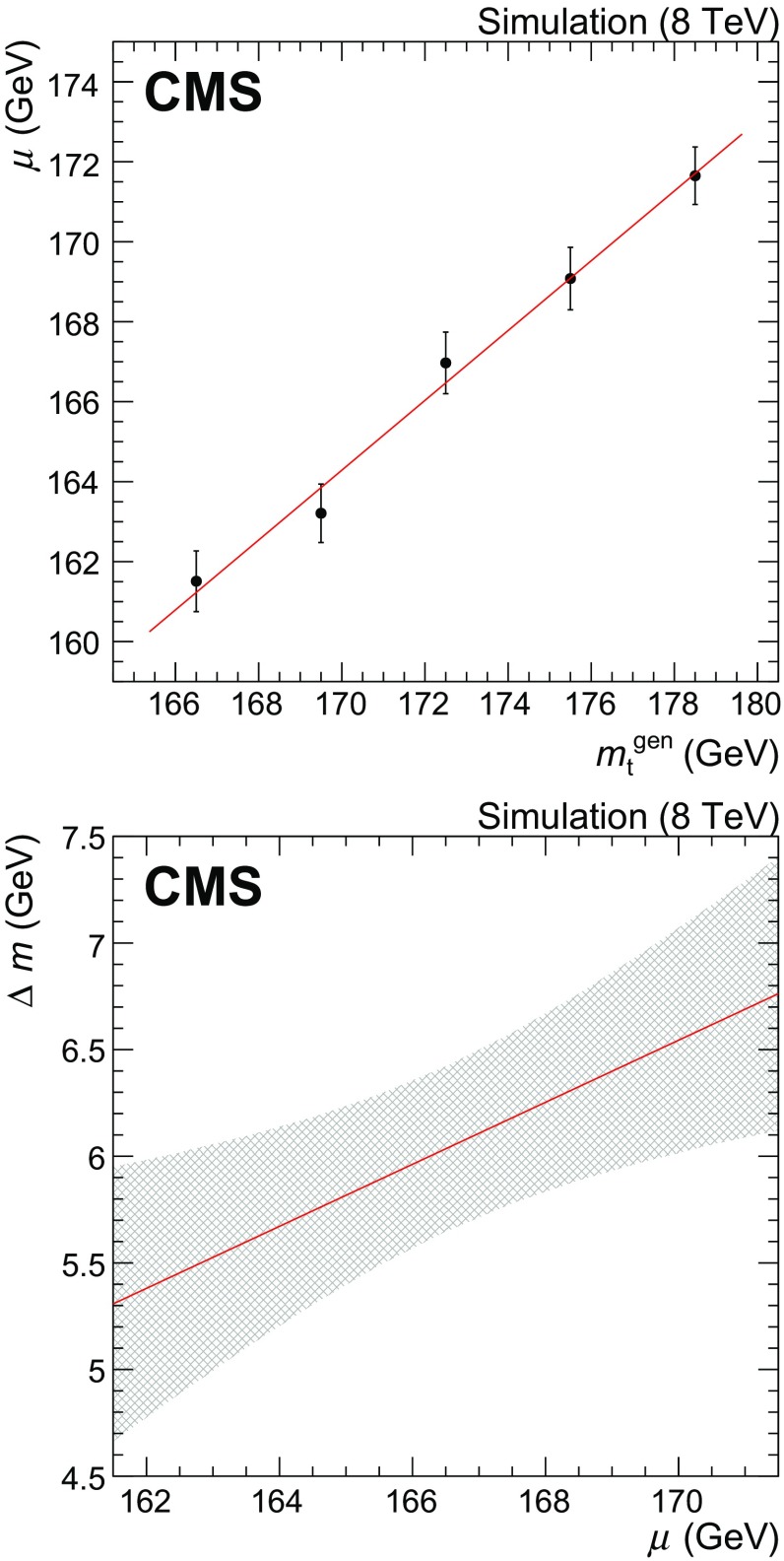
Mass calibration from fits to samples with different generated top quark mass. (*Top*) fit results as a function of the generated top quark mass. The *straight line* shows the result of a linear fit to the chosen top quark mass values. (*Lower*) mass shift, as a function of the fitted top quark mass (*straight line*). The *shaded grey area* represents the associated systematic uncertainty

The parameter $\mu$ of the Crystal Ball function describing the single top quark *t*-channel component is used to estimate the top quark mass. The mass is obtained by shifting the value of $\mu$ resulting from the fit by an amount $\Delta m$ depending on $\mu$ itself. In order to calibrate the magnitude of the shift, the fit has been repeated on a set of simulated samples including all signal and background processes, where the *t*-channel single top quark and $\mathrm{t}\overline{\mathrm{t}}$ events were generated with different values of the top quark mass, all other events remaining unchanged. Figure 5 shows the resulting values of $\mu$ as a function of the generated top quark mass (upper) and the mass calibration curve from a fit to these values (lower). The $\Delta m$ shift to be applied to the fitted value of $\mu$ is expressed as a linear function of $\mu$ itself. The shaded grey area represents the uncertainty associated with $\Delta m$, obtained from the statistical uncertainties of the fits.

### Parameterisation of the non-top-quark background

**Fig. 6:**
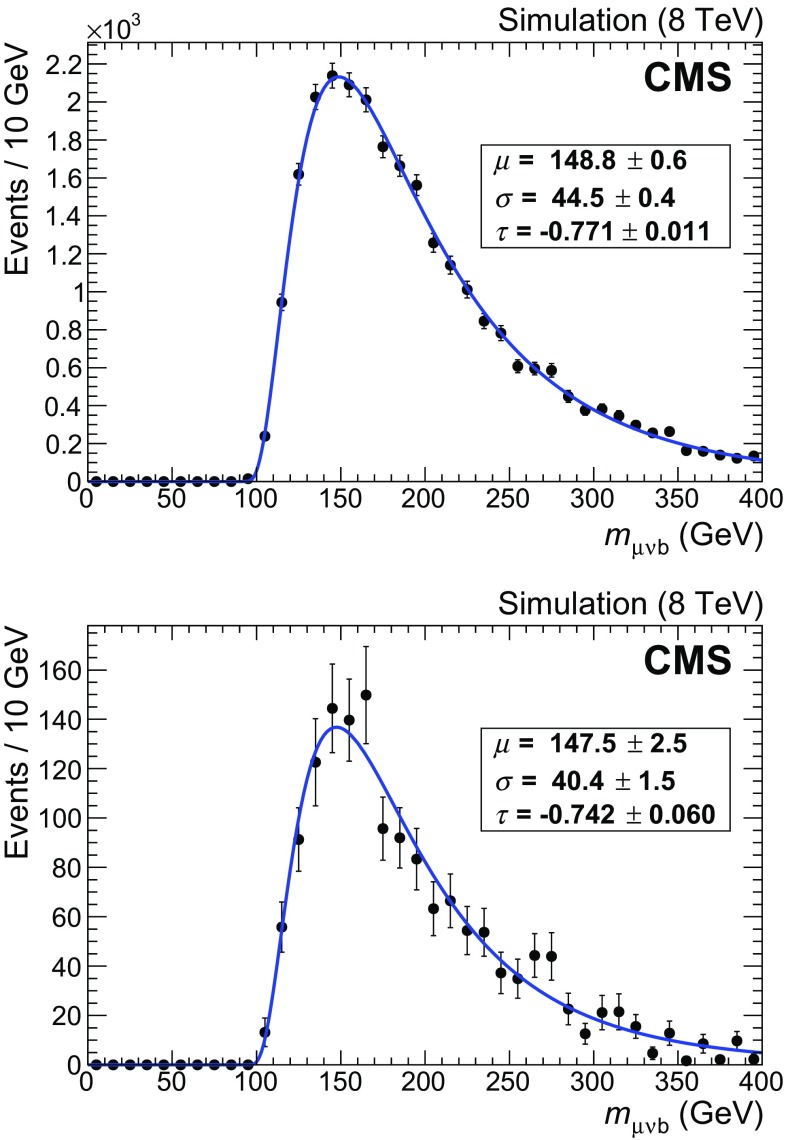
Reconstructed $\mu \nu \mathrm{b}$ invariant mass for non-top-quark background events, from Monte Carlo simulation. (*Top*) before final selection; (*lower*) after final selection. The *continuous lines* show the results of fits to Novosibirsk functions

The $\mathrm {W}$+jets events are expected to provide the largest contribution to the residual background. The ‘2J0T’ sample is mostly populated by such events and contains a large number of events, making it in principle a suitable control region to study the expected contribution of $\mathrm {W}$+jets events to the background in the signal region. However, the simulation shows that the reconstructed invariant mass distribution for $\mathrm {W}$+jets events in the ‘2J0T’ sample differs from that of the ‘2J1T’ sample. Thus, simulation has been used for the characterisation of the $\mathrm {W}$+jets component, as well as for all other non-top-quark background contributions. The shape of the invariant mass distribution for the sum of all non-top-quark background sources is well reproduced by a Novosibirsk function [37], with parameters $\mu$ and $\sigma$ representing the Gaussian core, and $\tau$ describing the skewness of the distribution. The option to use the full simulated sample before the final selection, as is done for events containing top quarks, has not been chosen, as the parameters of the fitted function vary significantly with the requirement on $|\eta _{\mathrm {j}'} |$, as shown in Fig. 6. Therefore, the sample obtained after applying the final selection is used to determine the shape parameters in the final fit.

### Determination of the top quark mass from the fit

The invariant mass distribution of the selected top quark candidates is fitted with three components corresponding to signal, $\mathrm{t}\overline{\mathrm{t}}$ and non-top-quark processes, using the probability density functions described above. The mass is obtained from the resulting value of the mean of the Gaussian core of the Crystal Ball function fitting the single top quark contribution, applying the calibration procedure described above. All parameters of the single top quark component are left free in the fit. The difference between the peak position of the *t*-channel and $\mathrm{t}\overline{\mathrm{t}}$ components is kept fixed to the value measured in simulation, to reduce the statistical fluctuations due to the small number of residual $\mathrm{t}\overline{\mathrm{t}}$ events. All remaining parameters (including normalisations) are fixed to the values extracted from simulation, after applying the final event selection.

**Fig. 7:**
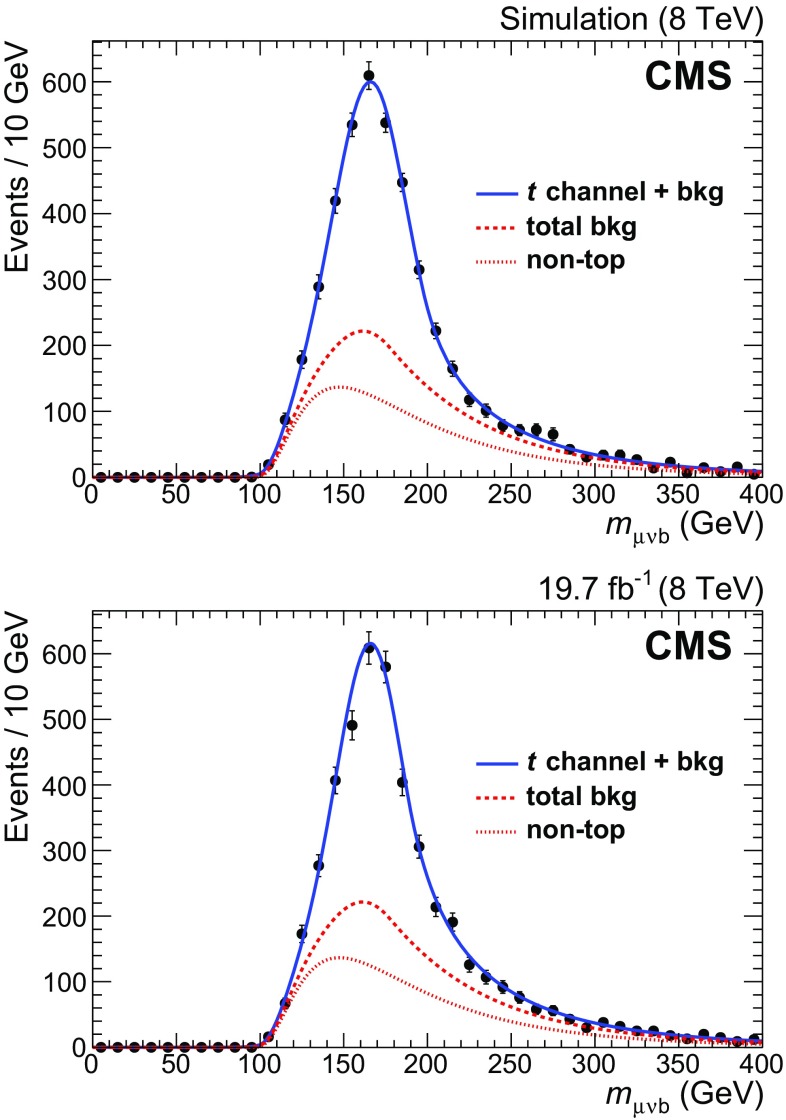
Result of the fit to the reconstructed $\mu \nu \mathrm{b}$ invariant mass. *Top* Monte Carlo simulation; *lower* data. In each plot, the *solid line* represents the result of the full fit; the *dotted line* shows the non-top-quark component, while the *dashed line* shows the sum of all background components

The results of the fits to the simulated sample and to the collision data sample are shown in Fig. 7. The number of *t*-channel events returned by the fit is $N_{\text {t-ch}}^{\text {fit}} = 2188 \pm 72$, in agreement with the number expected from simulation, $N_{\text {t-ch}}^{\mathrm {MC}} = 2216^{+94}_{-78}$. A value of $m_{\mathrm{t}} = 172.95 \pm 0.77\,\text {(stat)} \,\text {GeV}$ is obtained after applying the mass calibration (Fig. 5). A systematic uncertainty of $0.39\,\text {GeV}$ is associated to the mass calibration procedure.

### Cross-checks

The consistency and stability of the fit are assessed using pseudo-experiments. Ensembles of experiments are simulated using the signal and background templates, with their normalisations distributed according to Poisson statistics. In each pseudo-experiment, the same fit described above is repeated and the top quark mass and the signal yield are derived. The resulting distributions of the top quark mass and its root-mean-square show that the fit does not have any significant bias, with the difference between fitted and generated top quark masses, normalised to the fitted mass uncertainty (“pull”), distributed as expected.

Additionally, a test has been made where both the single top quark contribution and the $\mathrm{t}\overline{\mathrm{t}}$ components are fitted with a single Crystal Ball function. The results do not change appreciably within the present uncertainties with respect to the nominal fit.

The mass measurement for the single top quark contribution is derived after having removed the single top antiquark events. As a check, the analysis has been repeated and the top quark mass has been measured using single top antiquark events. The difference between the two measurements is $0.8 \pm 1.2\,\text {GeV}$, with a difference of $-0.6 \pm 1.5\,\text {GeV}$ expected from simulation. Furthermore, the fit has been performed by simultaneously fitting single top quark and single top antiquark candidates: the fitted mass does not statistically differ with respect to the result obtained with the nominal fit. These studies confirm that the selection of only the top quark candidates does not introduce any bias in the measured top quark mass.

## Systematic uncertainties

Many of the uncertainties described below use modifications of the simulation to assess the impact on the final result. These modifications affect the shapes and normalisations of the templates used by the fit. Their contributions have been evaluated following the strategy adopted in Ref. [38]: the uncertainties are categorised consistently to allow effective combinations with other top quark mass measurements.

In the following, the sources of uncertainties identified as relevant for the measurement are described, as well as the procedure adopted to evaluate their impact. All the uncertainties are then combined in quadrature to derive the total systematic uncertainty.

- *Jet energy scale (JES):* JES factors are applied to the jet energy response in simulation to match that observed in data. The JES uncertainties are $p_{\mathrm {T}}$- and $\eta$-dependent, and are taken into account by scaling the energies of all jets up and down according to their individual uncertainties, as determined in dedicated studies [31]. The scaling is then propagated to the calculation of $p_{\mathrm {T}} ^\text {miss}$, and all other quantities dependent on the jet energies. The mass fit is repeated on the ‘scaled’ simulated sample and the shift with respect to the nominal fit is taken as a measure of the uncertainty. The uncertainties in the JES are subdivided into independent sources and grouped into different categories following the prescription defined in Ref. [39], aimed at simplifying the combination of measurements reported by the different LHC experiments. A total of five categories are identified referring to the effect of uncertainties related to the absolute scale determination using Drell–Yan events (“in-situ correlation group”), relative ($\eta$-dependent) calibration, and high- and low-$p_{\mathrm {T}}$ extrapolation (“inter-calibration group”), flavour-specific corrections (“flavour-correlation group”), pileup corrections using an offset dependence on the jet $p_{\mathrm {T}}$ (“pileup $p_{\mathrm {T}}$ uncertainty”), and remaining sources, uncorrelated between ATLAS and CMS (“uncorrelated group”).
- *b quark hadronisation model:* This is the term that accounts for the flavour-dependent uncertainties arising from the simulation of the parton fragmentation. The total uncertainty can be decomposed into two separate contributions: the $\mathrm{b}$ quark fragmentation uncertainty and the uncertainty from $\mathrm{b}$ hadron decays. The b quark fragmentation uncertainty has been derived in the same way as in the top quark mass measurement using semileptonic $\mathrm{t}\overline{\mathrm{t}}$ events [38]. The Bowler–Lund fragmentation function for $\mathrm{b}$ hadrons is retuned to agree with the $x_{\mathrm {B}}$ data measured by the ALEPH [40] and DELPHI [41] Collaborations, where $x_{\mathrm {B}}$ represents the fraction of the $\mathrm{b}$ quark energy retained by a $\mathrm{b}$ hadron. A weight is attributed to each event, according to the $x_{\mathrm {B}}$ value, and the difference with respect to the nominal setup is taken as the systematic uncertainty. The systematic uncertainty from the semileptonic branching ratio of $\mathrm{b}$ hadrons is taken from Ref. [38], in which the branching fractions were varied by $-0.45$ and $+0.77\%$ to give the possible range of values and the associated uncertainty.
- *Jet energy resolution (JER):* After correcting for the mismatch between the data and simulation for the energy resolution, the uncertainty is determined by varying the corrected JER within its $\eta$-dependent ±1 standard deviation uncertainties. These changes are then propagated to the calculation of $p_{\mathrm {T}} ^\text {miss}$.
- *Muon momentum scale:* This contribution is determined by varying the reconstructed muon momenta by their uncertainties. These are determined as a function of the muon $\eta$ and $p_{\mathrm {T}}$ with a “tag-and-probe” method based on Drell–Yan data, as described in Ref. [42].
- *Unclustered missing transverse momentum:* The uncertainty arising from the component of the missing transverse momentum that is not due to particles reconstructed as leptons and photons or clustered in jets (“unclustered $p_{\mathrm {T}} ^\text {miss}$ ”) is determined by varying it by ±10%.
- *Pileup:* This is the uncertainty coming from the modelling of the pileup in data. It is taken as the sum of the effect of the uncertainty in the pileup rate (evaluated with pseudo-experiments in which the effective inelastic pp cross section has been varied by ±5%) and the pileup term extracted from the JES ‘uncorrelated’ group (see above).
- *b tagging efficiency:* To calculate the uncertainties from the $\mathrm{b}$ tagging efficiency and the misidentification rate, the $p_{\mathrm {T}}$- and $\eta$-dependent $\mathrm{b}$ tagging and misidentification scale factors are varied within their uncertainties for heavy- and light-flavour jets, as estimated from control samples [32]. The resulting changes are propagated to the event weights applied to the simulated events to obtain the uncertainties.
- *Fit calibration:* The mass is derived from the value of $\mu$ returned by the fit according to the mass calibration procedure described before: the same procedure provides an associated systematic uncertainty (Fig. 5, lower).
- *Background estimate:* This is the uncertainty resulting from the use of simulated backgrounds in the mass determination. One contribution to the systematic uncertainty is determined by varying the background normalisations by ±1 times their standard deviation uncertainties. In addition, in the fit, the shape parameters of both the $\mathrm{t}\overline{\mathrm{t}}$ and the W+jets components are fixed: these parameters are varied by ±1 times their standard deviation uncertainties. As there are theoretical uncertainties on the inputs to the simulation which may alter the background shapes used in the mass fit, an additional ‘radiation and matrix-element to parton-shower matching’ uncertainty is included, as described below.
- *Generator model:* Depending on whether the b quarks are considered part of the proton or not, the production of single top quarks can be studied in the 5- or 4-flavour schemes [10], respectively. The signal sample used in this work is produced with the 5-flavour scheme, where the b quarks are considered as constituents of the proton. To estimate the systematic uncertainty due to treating the b quarks like the lighter quarks, a comparison between a 5- and a 4-flavour-scheme ($2 \rightarrow 2$ and $2 \rightarrow 3$, respectively) samples has been performed: in the latter, the b quarks are generated in the hard scattering from gluon splitting. The samples used for the comparison are produced using the CompHEP generator [43], version 4.5.1, with the same configuration as the nominal signal sample.
- *Hadronisation model:* This uncertainty is already covered by the JES uncertainty and $\mathrm{b}$ quark hadronisation uncertainties considered above. As a cross-check, the nominal simulation is compared with results obtained using the herwig generator [44], version 6.520, tune AUET2 [45], for parton showering and hadronisation. The resulting difference is in agreement with what is obtained for the JES uncertainty.
- Radiation and matrix-element to parton-shower matching: This is the category which covers the QCD factorisation and renormalisation scale (with the nominal values of $\mu _{\mathrm {R}}=\mu _{\mathrm {F}}=Q^2$) and initial- and final-state radiation uncertainties. For the renormalisation and factorisation scale uncertainty determination, dedicated samples with $\mu _{\mathrm {R}}$ and $\mu _{\mathrm {F}}$ scales shifted up or down by a factor of 2 are used. The uncertainty is determined by comparing the central result with the shifted ones. For the matrix-element to parton-shower matching thresholds, $\mathrm{t}\overline{\mathrm{t}}$ and $\mathrm {W}$+jets samples in which the thresholds have been shifted up or down by a factor of 2 are used, with the systematic uncertainty evaluated in the same way as for the scale uncertainty. This is not relevant for the signal data set, which does not have a matrix-element to parton-shower matching. This procedure covers initial- and final-state radiation uncertainties. All variations are applied independently of each other and the corresponding uncertainties are treated as uncorrelated.
- *Underlying event:* This term represents the uncertainty coming from the modelling of the underlying event (UE), the particles from the interaction that do not enter into the hard parton-parton interaction. It is evaluated by comparing the results from two different tunes of pythia, the default Z2* tune and the Perugia tune [46]. The differences in the value of the fitted mass are within the statistical uncertainty determined by the size of the simulated samples. In fact, the two opposite variations result in mass shifts with the same sign. For this reason, the uncertainty from this source is estimated as the maximum statistical uncertainty of the changes.
- *Colour reconnection:* This uncertainty is evaluated by comparing two different UE tunes in which one has the nominal colour-reconnection effects and the other has these turned off.
- *Parton distribution functions:* The PDF4LHC [14] prescriptions are followed to calculate the uncertainty due to the choice of the PDFs. The variation of the fitted top quark mass is estimated by using alternative sets of PDFs with respect to the nominal one, namely the MSTW2008CP [17], CT10 [24], and NNPDF2.3 [15] sets.

The systematic uncertainties that affect the top quark mass measurement are summarised in Table 1. Sources of systematic uncertainties that are totally or partially uncorrelated with the top quark mass measurements using $\mathrm{t}\overline{\mathrm{t}}$ events are the fit calibration, the background estimate, the generator model and theoretical parameters for the simulation of signal events, and the colour-reconnection effects.

**Table 1:** Systematic uncertainties in the top quark mass

Source	Subcategory	Uncertainty ($\,\text {GeV}$ )
Jet energy scale	In-situ correlation group	$+0.20, -0.21$
	Inter-calibration group	${\pm }0.05$
	Flavour-correlation group	${\pm }0.40$
	Pileup $p_{\mathrm {T}}$ uncertainty	$+0.18, -0.10$
	Uncorrelated group	$+0.48, -0.40$
	Total	$+0.68, -0.61$
$\mathrm{b}$ quark JES and hadronisation model		${\pm }0.15$
Jet energy resolution		${\pm }0.05$
Muon momentum scale		${\pm }0.05$
$p_{\mathrm {T}} ^\text {miss}$		${\pm }0.15$
Pileup		${\pm }0.10$
$\mathrm{b}$ tagging efficiency		${\pm }0.10$
Fit calibration		${\pm }0.39$
Background estimate	Shape	${\pm }0.10$
	Normalisation	${\pm }0.14$
	$\mu _{\mathrm {R}}$ and $\mu _{\mathrm {F}}$ scales	${\pm }0.18$
	Matching scales	${\pm }0.30$
	Total	${\pm }0.39$
Generator model		${\pm }0.10$
Signal $\mu _{\mathrm {R}}$ and $\mu _{\mathrm {F}}$ scales		${\pm }0.23$
Underlying event		${\pm }0.20$
Colour reconnection		${\pm }0.05$
Parton distribution functions		${\pm }0.05$
Total		$+0.97, -0.93$

## Summary

The top quark mass is measured in a sample enriched in events with a single top quark for the first time. Top quarks are reconstructed from decays to a $\mathrm {W}$ boson and a $\mathrm{b}$ quark, with the $\mathrm {W}$ boson decaying to a muon and a neutrino. In the final sample, events with a top quark from single production in the *t*-channel account for 73% of the total number of events with a top quark. The measurement is obtained from a fit to the distribution of the reconstructed mass of top quark candidates, where the *t*-channel single top quark component is modelled separately from the contribution of other top quark production channels. The measured value is $m_{\mathrm{t}} = 172.95 \pm 0.77\,\text {(stat)} ^{+0.97}_{-0.93}\,\text {(syst)} \,\text {GeV}$. This is in agreement with the current combination of Tevatron and LHC results, $173.34 \pm 0.27\,\text {(stat)} \pm 0.71\,\text {(syst)} \,\text {GeV}$, which is based on measurements using $\mathrm{t}\overline{\mathrm{t}}$ events. Because many of the systematic uncertainties affecting the measurement of $m_{\mathrm{t}}$ using single top quark *t*-channel events are totally or partially uncorrelated with the measurements using $\mathrm{t}\overline{\mathrm{t}}$ events, and in addition the data sample analysed is largely statistically independent of the samples previously used, the result presented in this paper will be useful in the determination of world averages of the top quark mass.
